# Microbiota of the Therapeutic Euganean Thermal Muds with a Focus on the Main Cyanobacteria Species

**DOI:** 10.3390/microorganisms8101590

**Published:** 2020-10-15

**Authors:** Barbara Gris, Laura Treu, Raffaella Margherita Zampieri, Fabrizio Caldara, Chiara Romualdi, Stefano Campanaro, Nicoletta La Rocca

**Affiliations:** 1 Department of Biology, University of Padova, Via U. Bassi 58/B, 35121 Padova, Italy; barbara.gris@phd.unipd.it (B.G.); laura.treu@unipd.it (L.T.); raffaellamargherita.zampieri@phd.unipd.it (R.M.Z.); chiara.romualdi@unipd.it (C.R.); stefano.campanaro@unipd.it (S.C.); 2 Botanical Garden, University of Padova, Via Orto Botanico 15, 35123 Padova, Italy; 3 Pietro d’Abano Thermal Studies Center, Via Jappelli 5, 35031 Abano Terme, Italy; fabrizio.caldara@centrostuditermali.org; 4 CRIBI Biotechnology Center, University of Padova, 35121 Padova, Italy

**Keywords:** peloids, microbial community, cyanobacteria, *Phormidium* ETS-05, Euganean district, therapeutic thermal muds

## Abstract

The Euganean Thermal District has been known since Roman times for the therapeutic properties of peloids, obtained from natural clays that have undergone a traditional maturation process. This leads to the growth of a green microbial biofilm with Cyanobacteria and the target species *Phormidium* sp. ETS-05 as fundamental components for their ability to synthetize anti-inflammatory molecules. Currently, in-depth studies on the microbiota colonizing Euganean peloids, as in general on peloids utilized worldwide, are missing. This is the first characterization of the microbial community of Euganean thermal muds, also investigating the effects of environmental factors on its composition. We analysed 53 muds from 29 sites (Spas) using a polyphasic approach, finding a stable microbiota peculiar to the area. Differences among mud samples mainly depended on two parameters: water temperature and shading of mud maturation plants. In the range 37–47 °C and in the case of irradiance attenuation due to the presence of protective roofs, a statistically significant higher mud Chl *a* content was detected. Moreover, in these conditions, a characteristic microbial and Cyanobacteria population composition dominated by *Phormidium* sp. ETS-05 was observed. We also obtained the complete genome sequence of this target species using a mixed sequencing approach based on Illumina and Nanopore sequencing.

## 1. Introduction

**Figure 1:**
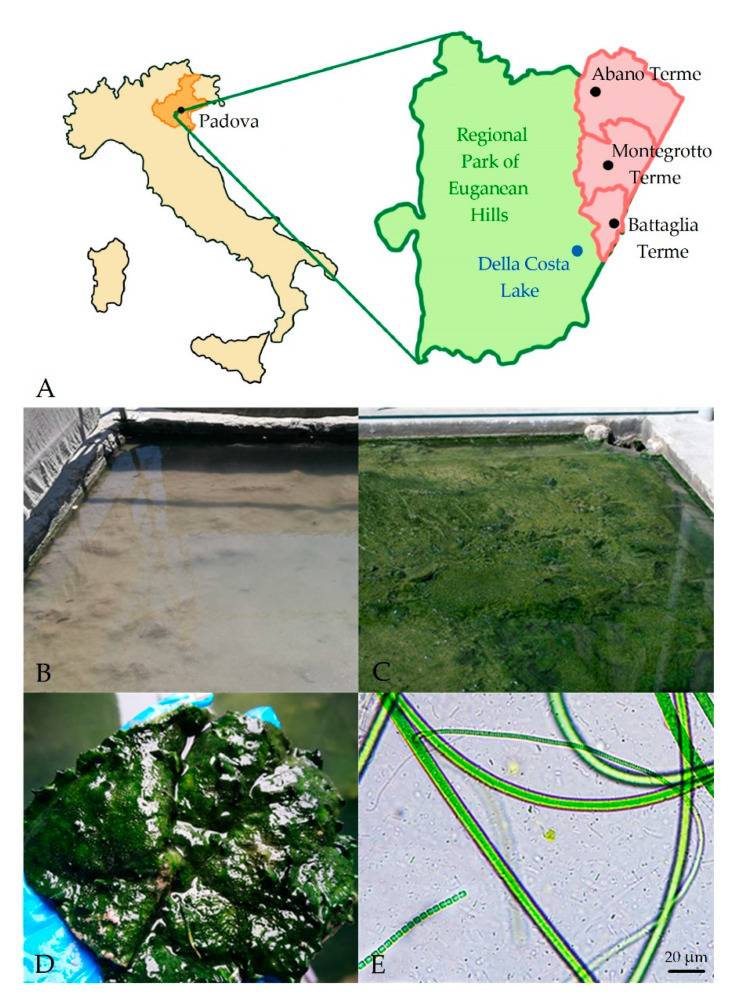
(**A**) Localization of the Euganean thermal district. Green represents the area of the Regional Park of the Euganean Hills, while the municipalities in which sampled sites are located are in red. (**B**,**C**) Example of a maturation pond at the beginning and at the end of mud maturation process. During maturation the virgin clay (B) is progressively colonized by a microbiota leading to the appearance on the mud surface of a biofilm with a typical green pigmentation (**C**) due to the presence of Cyanobacteria. (**D**) Portion of microbial biofilm collected from the mature mud surface and (**E**) image at light microscope of the microbial species colonizing it.

Peloids are special muds or clays consisting of mineral and organic components generated by geological and biological, chemical and physical processes. The use of peloids, for baths or packs, is called pelotherapy and shows beneficial effects in the healing of rheumatic disorders, osteoarthritis, skin diseases and other pathologic afflictions [1,2,3]. This is a common practice all over the world and has been used in Europe for 200 years. In most spas, peloids are obtained from natural muds and clays, mined in local lakes or seas, subjected to a maturation period of up to 2 years in special ponds [2,4]. The Euganean Thermal District, internationally renowned for its therapeutic peloids, is situated in the North-East of Italy. It includes the Euganean Hills of volcanic origin and the surrounding plain with the thermal localities of Abano, Montegrotto, Battaglia Terme and Galzignano (Figure 1).

The area is characterized by an underground hydrothermal basin with thermal waters flowing to the surfaces through natural springs and artificial wells [5,6]. The water, having an average temperature of 75 °C, is classified as ‘hyperthermal’ [5] and owes its heat to geothermal factors. The rainwater, rising from depth to the surface in the highly permeable area of the Euganean hills, acquires a slight radioactivity and salinity due to rock erosion [5,6]. This thermal water (pH 6.3–7.4) is considered hypersaline and shows high values of total dissolved solids (from 2.3 to 6 g/L) with chlorine, sodium, potassium, magnesium, sulphur, bromide, iodine and silicon dioxide as principal minerals [5,7]. The Euganean area has been known since ancient times for the beneficial effects of both thermal waters and muds on human health. This is well documented by the archaeological traces of thermal baths built in Roman times, also mentioned in the writings of Livy and Pliny the Elder. Nowadays, almost 100 spas make up the Euganean District one of the most important centers of thermal baths and mud treatments in the world, with about 1.8 million mud applications per year [7]. Euganean mud treatments, consisting of the application of warm muds directly on the skin of patients are recognized by the Italian Health System as therapies for healing rheumatic diseases, as arthritis and osteoarthritis [8]. Each spa has its own hot thermal well and it is responsible for independently preparing the therapeutic peloids according to the regional rule (BUR, 2015). These rules codify the traditional Euganean mud maturation procedures and must be followed by the thermal structures to obtain the “Mature Mud AOC” certification. The beneficial mud, in fact, is prepared by a semi-natural process, called ‘maturation’ [2,7,9], which implies, as raw material, the use of the ‘virgin clay’ collected from the bottom (about 11 m deep) of the natural Della Costa thermal lake (see Kaltenrieder et al., 2010 and Calderan et al., 2020 for details [7,10]). This clay is laid in artificial ponds (generally 10–12 m^2^ in surface and 1–2 m in depth) or tanks (about 2–3 m in diameter and 2 m in depth) (Figure 1 and Figure S1) of the different thermal spas. The clay is initially moistened for few days with thermal water at over 55 °C, to eliminate undesired non thermophilic microorganisms and then maintained constantly covered by a layer of flowing water at a temperature around 40 °C for a period of at least two months (BUR, 2015). During this time, the growth of a microbial mat between the mud surface and the flowing water is always observed (Figure 1). The microorganisms, mainly represented by Cyanobacteria [8], are embedded in a thick polysaccharidic matrix and at the end of the process generate a green biofilm that indicates a correct mud maturation (Figure 1). The analysis of the mud’s chlorophyll *a* (Chl *a*) content is conventionally utilized as practical quality control of mud maturation (BUR, 2015). Finally, the mature mud is mixed and put in ponds with thermal water at nearly 60 °C (6–24 h), to reduce the natural microbial load and to maintain the fluidity of the product before its use for therapies. Pelotherapy is a diffuse practice in many spas all around the world and in several cases, as in the Euganean District, implies the use of natural clay subjected to maturation procedures leading to the enrichment of the mineral substrates with organic compounds released by microorganisms [2,9,11]. However, the main factors responsible for mud therapy’s beneficial effects are in general ascribed to mechanical, thermal and chemical properties [12,13], while only a few studies have partially characterized the microbiota of peloids and correlated its presence with mud therapeutic activities [2]. The Euganean District is one of the most studied sites and already represents a reference for the research in this field [2]. The development of green microbial mats on mature muds, that is a peculiarity of Euganean peloids, has been linked to the use of the local ‘virgin clay’ and to the application of the specific maturation process. Mud maturation experiments carried out comparing the natural substrate with commercial ones (such as bentonite) showed in fact a strong impairment of the green biofilm growth in the second case [14]. Moreover, surveys on some of the Cyanobacterial species colonizing the Euganean muds also demonstrated their ability to produce bioactive molecules with anti-inflammatory and antioxidant properties [15,16,17], tested both in vitro [18,19,20] and in vivo [21,22]. The isolated *Phormidium* sp. ETS-05 (ETS, Euganean Thermal Springs) is the most abundant strain present in the Euganean mature muds [23] and is considered the target species for the maturation process. Studies on the anti-inflammatory effectiveness of its lipidic compounds also led to obtaining a European Patent (EP1571203) on the therapeutic efficacy of Euganean peloids [24]. The microbiota biodiversity of thermal muds is, however, complex and not deeply characterized so far. A wide screen of photosynthetic microorganisms, based on microscopy observations, was published in 1975 [25], while studies on polyphasic characterizations of isolated Cyanobacteria are more recent [26,27]. No data are available regarding the unculturable microorganisms composing the Euganean muds community. Moreover, no information is published on the effects of environmental and operational conditions on the maturation process applied by the spas and on the stability of the mud microbiota. In this work we analyzed the effects of the physico-chemical parameters of thermal waters as well as of the presence of shading and protecting roofs on the Chl *a* content of several mature muds. The Chl *a* content is considered a good proxy of the Cyanobacteria amount in the mud and it was always considered for the evaluation of mud maturation level [14,15]. We also performed, for the first time, a characterization of the entire mud microbial community using high-throughput sequencing of the V4 hypervariable region of 16S rRNA, comparing molecular results with microscopy observations to better characterize the Cyanobacterial population. Furthermore, the complete sequence of the genome of *Phormidium* sp. ETS-05 was obtained using a hybrid approach based on Illumina and Nanopore sequencing.

## 2. Materials and Methods

### 2.1. Study Site and Sample Collection

The study was carried out on different Spas of the Euganean Thermal District, in the North-East of Italy (Figure 1A). Thermal waters and mature mud samples were collected in May 2017 and in May 2018 at the end of the mud maturation process (Figure 1) from a total of 53 mud maturation ponds or tanks of 29 thermal Spas (Table S1), 17 located in the municipalities of Abano Terme (45°21′42.84″ N 11°47′32.64″ E), 11 in Montegrotto Terme (45°20′ N 11°47′ E) and 1 in Battaglia Terme (45°17′21.73″ N 11°46′46.52″ E). For each selected pond or tank (Table S1), temperature (T), pH, electric conductivity (EC) and total dissolved solids (TDS) of water covering the mature mud, were measured using a multiparametric probe (Gro-Line HI 9814, Hanna Instruments, Padova, Italy). Light intensity and spectra quality reaching maturation systems were measured using a light-meter (LI-COR LI-180 Biosciences, Cambridge, UK). From the same sites, at least 20 subsamples of surface mature mud (1.5 cm depth) were harvested using sterile corers and then mixed in sterile containers to have representative final samples for chlorophyll content determination [14] and molecular analyses. Containers were maintained in a refrigerated box to rapidly reach the laboratory where mud samples were aliquoted and processed. Among all these samples, nine representatives were selected for DNA extraction and further characterization of the microbiota by next-generation sequencing (NGS) analyses. Moreover, at each site, microbial biofilms of the mature mud surfaces were collected by sterile pipettes in six randomly selected points, mixed in sterile tubes and utilized for microscopy analyses.

### 2.2. Pigments Extraction and Chlorophyll a (Chl a) Quantification

About 10 g of fresh mature mud samples of each sampling site were dried for 24 h in the dark at 40 °C [15], reduced to a fine powder in a mortar and used for chlorophyll *a* (Chl *a*) content determination. Pigments were extracted from 3 subsamples (0.5 g) of dry mud of each pond or tank. The mud powder was dissolved in 1 mL of N, N-dimethylformamide and incubated at 4 °C in the dark for 48 h. Samples were then centrifuged at 3000 g (Centrifuge MPW-352R, MPW Med. Instruments, Warsaw, Poland) for 10 min and the supernatant was analyzed using a spectrophotometer (Cary Series ultraviolet visible (UV-Vis), Agilent Technologies, Santa Clara, CA, USA), recording the absorption spectrum from 350 to 750 nm. The Chl *a* concentration of the extracts was determined using the specific extinction coefficient proposed by Moran [28] and considering the absorbance at 664 nm after subtraction of the value at 750 nm, that accounts for the turbidity. The Chl *a* content was then normalized on dry mud weight and calculated according to the following equation:

Chl *a* (µg∙g_MUD_^−1^) = (A_664_ − A_750_) × 11.92 × g_MUD_^−1^

### 2.3. DNA Extraction, Amplification and Sequencing

For nine selected sites, three aliquots (1.5–2 g) of fresh mature mud samples were frozen in liquid nitrogen and stored at −80 °C for molecular analyses. The total genomic DNA was extracted from samples in biological duplicates using a PowerSoil DNA Extraction Kit (MoBio laboratories, Carlsbad, CA, USA) following the manufacturer’s instructions. The early stages of the protocol were modified by adding a further purification step performed with 2 mL of phenol:chloroform:isoamyl alcohol (25:24:1) at pH 8 (Sigma Aldrich, Saint Louis, MO, USA) to clean samples from the bulk sediments. The DNA quantification and quality assessment were performed using NanoDrop (ThermoFisher Scientific, Waltham, MA, USA). Genomic DNA was aliquoted, freeze dried and shipped to Ramaciotti Centre for Genomics (University of South Wales, Sydney, Australia) for DNA sequencing. The V4 region of the 16S rRNA gene [29,30] was amplified using universal primers (bacterial and archaeal) 515F-806R [31] and amplicons were paired-end sequenced (2 × 150 bp) using MiSeq system (Illumina, San Diego, CA, USA). The raw reads were deposited in the sequence read archive database (SRA) of NCBI under the BioProject PRJNA624369.

### 2.4. Sequencing Data Analysis

Raw sequencing data were processed using CLC Workbench software (V.8.0.2) with the Microbial genomics module plugin (QIAGEN Bioinformatics, Hilden, Germany). Standard quality filtering (i.e., raw reads trimming), OTUs (operative taxonomic units) clustering, taxonomic assignment (Greengenes v13_8 database, Second Genome, Inc., Brisbane, CA, USA) alpha diversity in the form of rarefaction curves (Figure S2A) and beta diversity (i.e., PCoA) calculations were performed as previously described [32,33]. Briefly, once passing the filtering step the high-quality reads were clustered using the similarity percent specified by the OTU database (i.e., 97%) with de novo creation of new OTUs allowed. The alignment score for merging paired reads was left as default and final alignment results are reported in Figure S2B. Consensus sequences of most abundant OTUs (relative abundance >0.5% in at least 1 sample) were manually curated and compared with sequences in the GenBank database (http://www.ncbi.nlm.nih.gov/, accessed on 16 June 2020) using BLAST similarity search (nucleotide collection and 16S ribosomal RNA sequences databases), in order to validate the results. The consensus sequences of the abundant OTUs were manually verified and only the univocal hits to specific taxa according to recognised thresholds [34] were retained and used for OTUs’ nomenclature. An ETS (Euganean Thermal Springs) code was associated to each OTU for tracking purposes. ETS codes were further defined using numbers ranging from 1 to 100 for Cyanobacteria taxa, from 101 to 200 for Chloroflexi, from 201 to 300 for Proteobacteria and from 301 to 400 for Bacteroidetes. Heat maps and hierarchical clustering were done for main phyla, recalculating the relative abundances on each single phylum, visualized using the Multiple Experiment Viewer software (MeV 4.9.0) with complete linkage Pearson correlation, considering only OTUs with relative abundance >0.5%.

### 2.5. Light and Fluorescence Microscopy Analyses

For microscopy observations, two replicates of each collected sample were prepared and a dilution (1:20, *v*/*v*) with sterile thermal water was performed in order to remove most of the sediment before analyses. Cyanobacteria species were observed in a 50 µL-volume Nageotte cell counting chamber [35,36]. Cyanobacteria were divided in families or genera according to their morphology [37,38,39,40,41]. Using a 20× objective, about 20 fields were analysed and images were acquired using both visible and fluorescence lights (Leica DM 5000 B, Leica, Wetzlar, Germany), this last in order to check the cell viability and distinguish cells with coccoid morphology from mud sediments. Higher magnification objectives (40×, 63×) were used to collect more detailed images of species observed in samples. Images were then analysed using the open source ImageJ2 software (version 1.53e, National Institutes of Health, Bethesda, MD, USA) [42]. The dimension of a pixel in the images from the 20× objective was 9 µm; this value was used to calibrate the program. Before image processing, ‘area’, ‘perimeter’ and ‘length’ were set from ‘set measurements’ as morphometric parameters to be measured during image analysis. Images collected from the same field at different focal planes were simultaneously analysed using the ‘ROI (Region of Interest) manager’ function, which allows to maintain measurements done in all images opened at the same time. This is particularly useful in the case that it was not possible to focus on a filament in a single image. For filamentous species, areas were measured using the ‘Polygon selection’ tool, through manual contouring of filaments edges. From these values, the biovolumes were calculated considering filaments as cylinders [43,44]. Coccoid species were divided in groups based on cell size; the average biovolume of each group was calculated on a representative number of cells (*n* = 30), considering spherical and prolate spheroid shapes [43]. The biovolume of each group was calculated referring the average volume to the total cell number counted [44]. The biovolume of all coccoid species was calculated by adding the volumes of the individual groups. Population compositions of mud samples were calculated referring volumes of each morphogenera to the total Cyanobacteria volume [43]. A heat map was compiled from microscopy data using Multiple Experiment Viewer software (MeV 4.9.0, The Institute for Genomic Research, Rockville, MD, USA).

### 2.6. Phormidium sp. ETS-05 Cultivation, Genome Sequencing and Data Processing

The original strain of *Phormidium* sp. ETS-05 [23] was already present in our laboratory due to the collaboration with the Pietro d’Abano Thermal Research Center and cultivated in sterile liquid BG11 medium [45] in flasks. Temperature was maintained constant at 30 ± 1 °C and continuous light at an intensity of 20 µmol photons m^−2^ s^−1^ was provided. Three replicates of 7 mL of culture in exponential growth phase (OD_750_ = 0.4) were centrifuged in sterile tubes at 5000 g for 20 min in order to remove the supernatant. DNA extraction protocol was applied on cellular pellet using the same protocol described previously for mud samples. The sequencing was performed at the sequencing facility of the CRIBI Biotechnology Center (Padova, Italy) using a combined strategy that used Oxford Nanopore MinION single-molecule and Illumina NextSeq platforms. Libraries were prepared using SQL-RBK004 Rapid kit (Oxford Nanopore Technologies, Oxford, UK) for Oxford Nanopore and using Nextera DNA Flex Library Prep Kit (Illumina Inc., San Diego, CA, USA) for Illumina. The former was sequenced using FLO-MINSP6 on a MinION Mk 1B. The latter was analyzed using a NextSeq 500 Reagent kit v2 (150 cycles) for sequencing on a NextSeq 500 (Illumina Inc., San Diego CA) platform with a paired-end protocol. Base-calling on Oxford Nanopore raw data was undertaken using Guppy v2.3.7 [46], while high quality Illumina reads obtained by filtering using Trimmomatic (v0.39-1). The complete genome sequence was recovered with a stepwise procedure: first, Oxford Nanopore reads were assembled alone using minimap2 (v2.17-r941), miniasm (v0.3-r179) and minipolish (v0.1.2) (Li, 2018); second, Nanopore assembly was corrected using Illumina reads using a package of the MaSuRCA genome assembler (v3.3.7-0) [47]. Gene finding and annotation were performed using PROKKA (v1.12) [48] and the Rapid Annotation using Subsystem Technology (RAST) database [49]. Manual curation was applied in order to refine the lipid metabolism and the associated genes. Origin of replication (ORI) was identified and positioned in the genome sequence considering the sequence “TTTTCCACA” obtained from literature [50] and using it as an input for Ori-Finder 2 [51]. The four scaffolds obtained from the assembly, having 4.36, 1.43, 0.45 and 0.02 Mbp of length respectively, were ordered using MeDuSa scaffolder (v1.6-1, Florence computational biology group, University of Florence, Italy) [52] and merged using as a reference the closest genome available in the database, i.e., *Oscillatoria acuminata* PCC 6304 (GCF_000317105.1). The single-scaffold resulting from this process was represented as a circular genome using Artemis (v18.1.0, Sanger Institute, Saffron Walden, UK) [53], with a custom perl script for the clusters of orthologous groups (COG) classification [54] and Artemis (v18.1.0). EGG (evolutionary genealogy of genes) functional annotation was performed using the KEGG (Kyoto Encyclopedia Genes and Genomes) Mapper, Reconstruct Pathway tool [55]. Properties of the final genome sequence were determined using QUAST (v5.0.2) [56]. The sequences of 16S rRNA gene were extracted from the genome using a custom perl script [57] and the similarity with the non-redundant NCBI (nrNCBI) public database was determined. All public microbial genomes belonging to the Cyanobacteria phylum have been downloaded from the NCBI database (n:1471, 10 June 2019) and a local database was established. Proteins belonging to these genomes were predicted using Prodigal (v2.6.3) [58] and 400 phylogenetically informative marker proteins were used to generate a phylogenetic tree using PhyloPhlAn (v0.99) [59]. From the total Cyanobacteria, a small subgroup of 76 species including *Phormidium* sp. ETS-05 has been selected using Dendroscope (v2.7.4) [60] and analyzed using dRep (v2.3.2) [61]. Average nucleotide identity (ANI) was calculated to evaluate the similarity level among genomes [62] (Figure S3) The genome has been deposited at NCBI microbial genomes database as *Phormidium* sp. ETS-05 under the BioProject PRJNA622676.

### 2.7. Statistical Analyses

Correlations between Cyanobacteria genera and water parameters were verified using Pearson’s correlation index r, calculated using R software (v 3.6.3, R Development Core Team, Auckland, New Zealand) [63]. Student’s *t*-tests were applied to ascertain significant differences in Chl *a* content at different conditions (water parameters and presence of protective roofs). The level of statistical significance was *p* < 0.05.

## 3. Results

### 3.1. Physico-Chemical Parameters of Thermal Waters and Chlorophyll a Content of Muds

**Figure 2:**
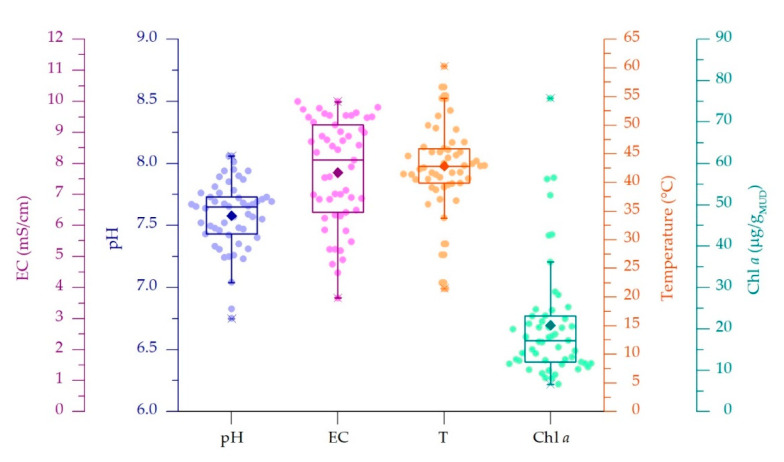
Distributions of data of thermal water physico-chemical parameters and of chlorophyll *a* content of mud samples. Boxplots showing average value (filled rhombus), median (horizontal lines), interquartile range (IQR) and 1.5 × IQR (with outliers indicated by X).

In the current study, 53 samples were collected from 29 thermal spas situated in Abano, Montegrotto and Battaglia Terme (Figure 1A). pH, electric conductivity (EC), total dissolved solids (TDS) and temperature (T) of thermal waters flowing on the mature mud surface were measured during mud sampling at two time points (May 2017 and 2018, Table S1). Solar radiation of the maturation period was retrieved from a weather station located in the municipality of Abano Terme (www.abanocity.it/meteo, accessed on 25 October 2018). The average irradiance of the 2 months preceding the sampling periods, accounting for the time of mud maturation, was 187 ± 15.7 W/m^2^. Considering that some spas have protective roofs covering the mud maturation systems, direct measurements of irradiance and light spectrum at the water surface level were monitored during sampling. Light intensity resulted in being attenuated up to 80–90%, depending on the shade generated by the presence of different types of covering systems. In these cases, no changes of the light spectrum were observed. Spas with the protective roofs are highlighted in Table S1. As indicated by the data distribution displayed in the boxplot graphs (Figure 2), the physico-chemical parameter values showed to be variable, with pH and EC showing average values of 7.58 ± 0.27 and. 7.7 ± 1.71 mS/cm, respectively. EC values showed large deviation from the average, mainly depending on the location of thermal water wells in the Euganean area. Lower EC values (from 4.47 mS/cm and 2250 ppm) were recorded for spas near the hills while higher EC values (up to 9.99 mS/cm and 5000 ppm) were in Abano Terme (Table S1). The water T was on average 42.9 ± 7.5 °C, with a large number of ponds and tanks tested falling in the range 39.9–45.9 °C (Figure 2), thus with values quite close to the 40 °C suggested by the regional rules for a correct mud maturation process. However, T data distribution also showed minimum and maximum values at 33.8 and 54.6 °C respectively, and two outliers at 21.5 and 60.3 °C. We investigated how these variations in environmental and operating conditions (water parameters and the presence of protective roofs) were affecting the mud maturation process and the development of the green microbial biofilm and the Cyanobacteria growth. This last aspect was assessed through the determination of the Chl *a* content of the mud, at surface and at one cm of depth. Chl *a* content was on average of 20.9 ± 14 µg/g_MUD_ with a median of 17.2 µg/g_MUD_, with minimum and maximum values of 6.6 and 36 µg/g_MUD_ respectively (Figure 2) and few outliers.

**Figure 3:**
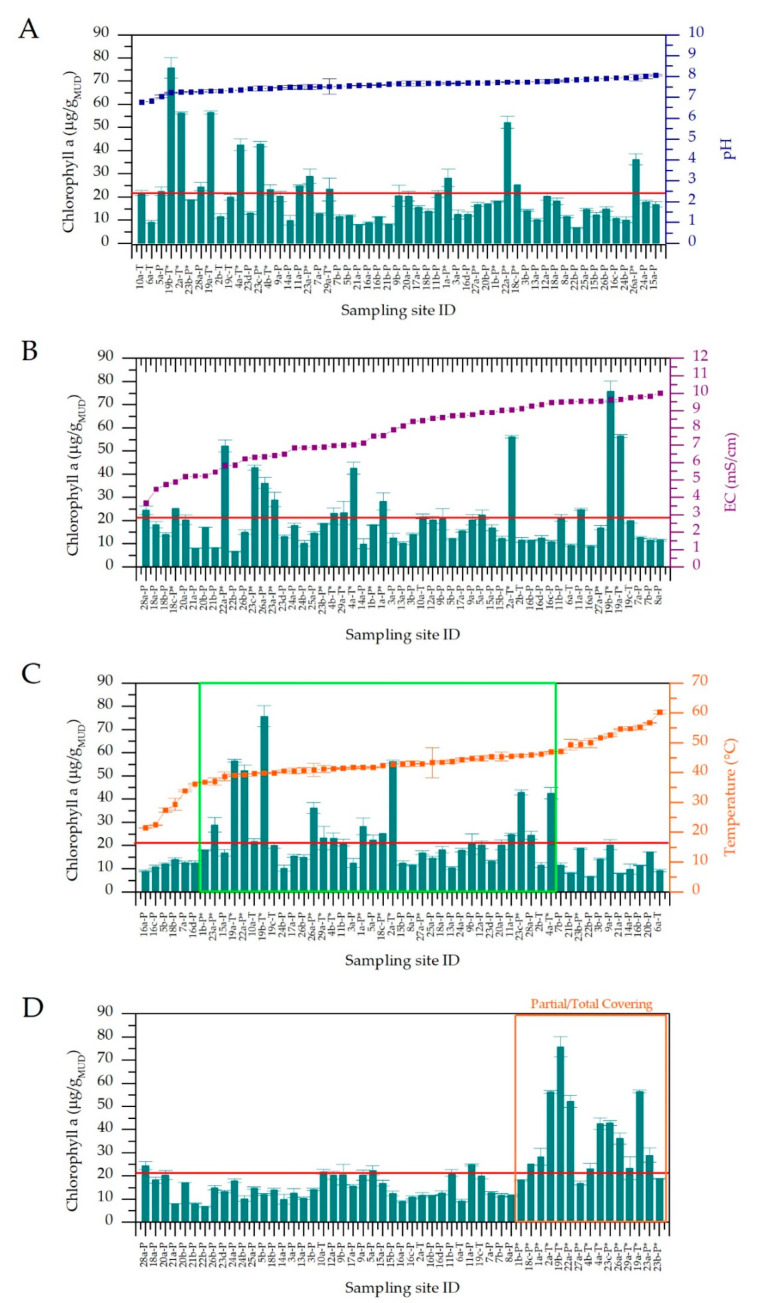
Chlorophyll *a* content of mature muds of the different spas as function of increasing values of pH (**A**), electric conductivity (**B**), temperature (**C**,**D**) sorted by the absence or presence of protective roofs (**D**). Red lines indicate the average Chl *a* content of mature muds at the end of maturation process. In figure C, the green box represents the central temperature range (37–47 °C). (Sample collected from maturation systems provided by protective roofs are indicated with *).

In Figure 3, the trends of Chl *a* mud content are reported together with pH (A), EC (B), T values (C) considering the presence of protective roofs over the maturation systems (D). Three temperature ranges, with statistically significant variation of data, were observed (Figure 3C). Below 37 °C and over 47 °C, the average Chl *a* mud content was considerably lower (11.8 ± 1.77 and 12.4 ± 4.90 µg/g_MUD_) than the average calculated on all samples (20.9 µg/g_MUD_). However, this value resulted significantly higher in the range 37–47 °C (24.7 ± 15.7 µg/g_MUD_) in respect to those obtained at water temperatures lower than 37 °C and higher than 47 °C (*p*-values of 0.041 and 0.009 respectively). Furthermore, in the central range, some spas showed Chl *a* values considerably higher than the total average. In these cases, maturation systems were provided by protective roofs, probably reducing the possible light stress effects of direct solar irradiation to the biofilms, allowing a faster growth of Cyanobacteria. The presence of protective roofs allowed the final Chl *a* values (36.3 ± 3.47 µg/g_MUD_) to double with respect to maturation plants completely exposed to solar radiation (14.8 ± 4.94 µg/g_MUD_), with statistically significant differences (*p*-value 5.88 × 10^−9^) (Figure 3D). Considering that almost all sampling sites with protection were found in the central range of temperatures, we applied a Student’s *t*-test on values from non-covered sampling sites; this was done to exclude the statistical significance of Chl *a* contents of muds in relation to temperature ranges being biased by these data. Results showed an average Chl *a* content of 16.8 ± 4.68 µg/g_MUD_ for sites in the range 37–47 °C, significantly higher than the average values found at temperature lower than 37 °C (11.8 ± 1.74 µg/g_MUD_, *p*-value 0.017), and higher than 47.0 °C (11.7 ± 4.63 µg/g_MUD_, *p*-value 0.008). In the same way, the influence of the presence of protective roofs on Chl *a* content was evaluated only in the central temperature range to avoid any interference of low Chl *a* values due to low or high temperatures. Results showed a statistically significant difference (*p*-value 1.29 × 10^−6^) from covered and uncovered sampled sites, with average Chl *a* content of 39.1 ± 3.47 and 16.1 ± 5.19 µg/g_MUD_, respectively. These results confirm that both temperature and the presence of protective roofs significantly affect the final mud’s Chl *a* content. The other physico-chemical water parameters showed no significant influence on this value, with *p*-values of 0.089 and 0.516 for pH and EC respectively (Figure 3A,B). In light of these results, we further correlate mud maturation water temperature and mud’s Chl *a* content with the community composition of the entire microbiota, with the Cyanobacteria community composition and biodiversity using a polyphasic approach (next-generation sequencing (NGS) and light microscopy).

### 3.2. Bacteria and Archaea Community Composition

**Figure 4:**
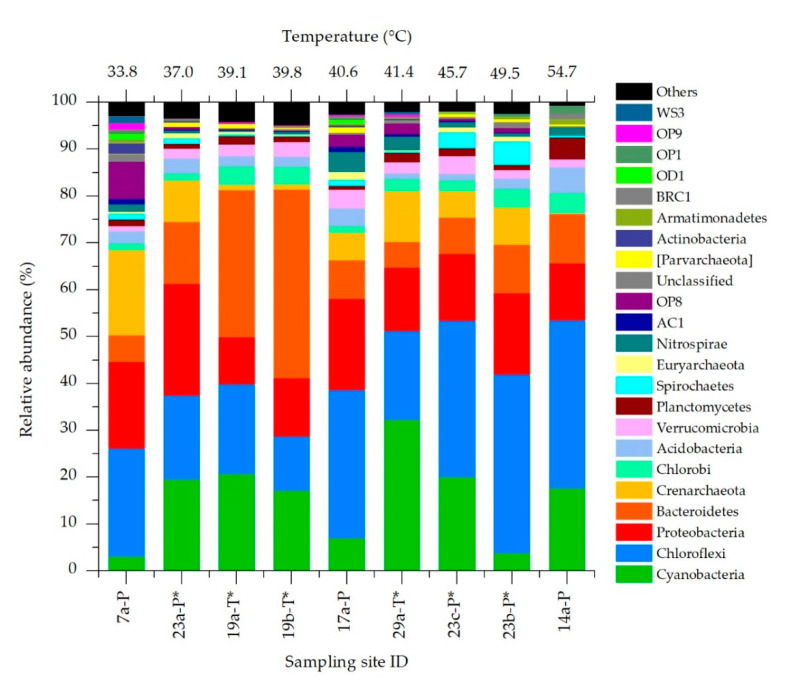
Relative abundance of the main phyla identified in the mature mud microbiota at different temperatures and sampling sites. Samples collected from maturation systems provided with protective roofs are indicated with *.

For molecular analysis we selected nine representative sampling sites considering water temperatures ranging from lower to higher values and mud collected from the same spa at different temperatures and from different spas at similar temperatures. Additionally, samples from both types of mud maturation systems (ponds and tanks) whether or not with protective roofs have been selected. The microbial community of each mud sample was investigated by high-throughput sequencing of the hypervariable region V4 of the 16S rRNA gene. The number of raw reads obtained per sample ranged between ~120,000 and ~190,000. According to the assessment of species richness (alpha diversity), the coverage was high enough to reach the saturation level and to obtain a complete overview of the taxa present (Figure S2A). At least 80% of raw reads were assigned to OTUs, whose number in the different samples ranged from 485 and 939. Shannon indexes revealed no significant differences in biodiversity or in association with environmental parameters. However, α-diversity tended to be higher in samples collected from ponds than tanks, with a *p*-value that, although showing no statistically significant difference (*p* = 0.0524), suggests a possible influence of the maturation system adopted on species biodiversity. Abundant OTUs represented less than 10% of the total diversity but they accounted for at least 60% of total community abundance (Figure S2B). The overall composition of the microbiome analyzed resulted similar among samples, with the presence of 4 main phyla (Chloroflexi, Cyanobacteria, Proteobacteria and Bacteroidetes) accounting from 50% to 81% of the total community (Figure 4). The 16S rRNA sequences of OTUs corresponding to the most abundant taxa and to all Cyanobacteria were reported in Table S2.

The most abundant phylum was Chloroflexi (25.5% on average), particularly at higher temperatures. Cyanobacteria showed an average relative abundance of 15.7%, and was very variable among the tested sites (3.07–32.3%). Proteobacteria and Bacteroidetes also shared similar average abundances (15.7 and 14.8% respectively), with the former showing quite constant values among samples. Despite these similarities, some outliers were observed, especially at the lowest and highest water temperatures. At 33.8 °C the relative abundance of Crenarchaeota and OP8 (*Candidatus aminicenantes*) phyla were higher than any other temperature. On the other side, at 54.7 °C the phylum Crenarchaeota showed a very low abundance (less than 1%), with higher presence of the phylum Planctomycetes. At medium-high temperatures (45.7–49.5 °C) the phylum Spirochaetes showed the maximum relative abundance.

### 3.3. Effects of Temperature on Chloroflexi, Proteobacteria and Bacteroidetes Communities Structure

The composition of the phyla Chloroflexi, Proteobacteria and Bacteroidetes was analyzed in detail (Figures S4–S6). The OTUs assigned to Chloroflexi (Figure S4) were characterized by a low taxonomic resolution, and most of them were assigned only at order or family level. Many OTUs were assigned to *Anaerolineales* or *Anaerolineaceae*, representing at least 47% of total abundance in almost all spas, up to 49.5 °C. Among them, *Anaerolineales* sp. ETS-102 was the most abundant, accounting on average for 22.8% of this phylum. Although Chloroflexi revealed an increase in relative abundance in samples collected at higher temperatures (>45.7 °C), only at 54.7 °C a different composition was observed, with the species *Roseiflexus* sp. ETS-120 being the most abundant. The OTUs assigned to the Proteobacteria (Figure S5) had higher taxonomic resolution than those previously described for Chloroflexi, revealing the presence of 11 genera and 10 species, with the most represented taxa showing a remarkable variability across different samples. The most abundant OTUs were *Thiobacter subterraneus* ETS-201 and *Thiobacter* sp. ETS-203, especially in samples collected from spas using ponds as maturation systems (average 18.8%). Samples collected from tanks showed peculiar taxa composition with specific groups colonizing muds. As regards the Bacteroidetes (Figure S6), *Haliscomenobacteraceae* was the most abundant taxon, with ETS-302 and ETS-301 being the most abundant OTUs in all samples, except for the lowest temperature, at which *Lewinellaceae* sp. ETS-303 was found as dominant. Only at 54.7 °C, an increase of *Schleiferia thermophila* ETS-305 and *Rhodothermaceae* sp. ETS-314 was observed.

### 3.4. Temperature Effect on Cyanobacteria Community

**Figure 5:**
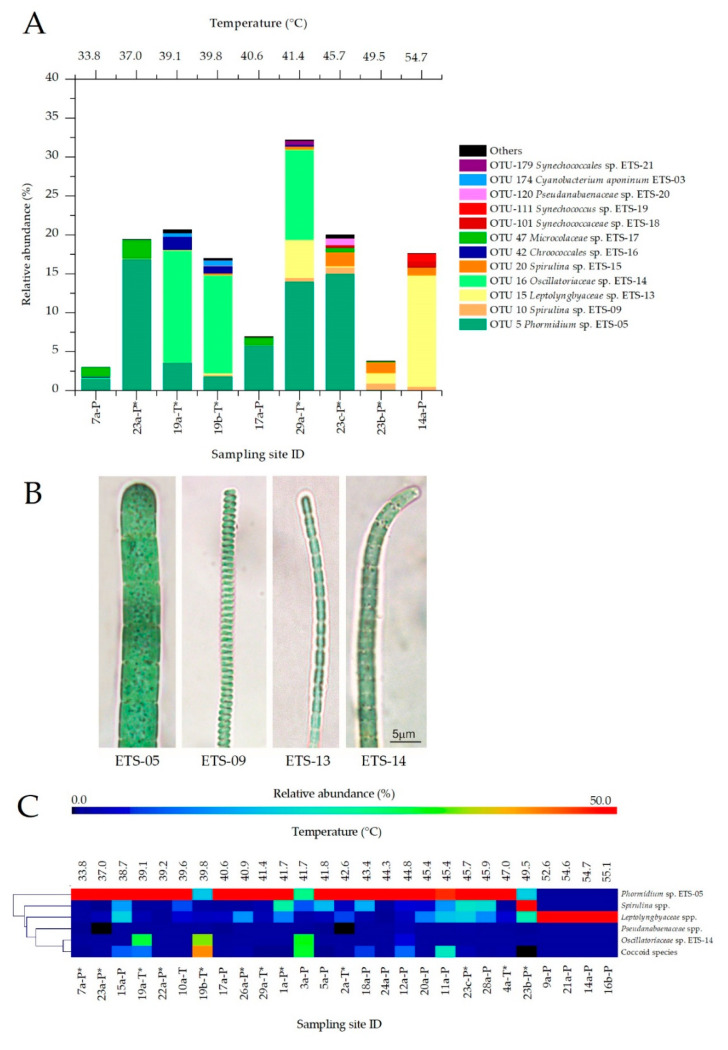
(**A**) Relative abundance of the main Cyanobacteria operative taxonomic units (OTUs) identified by next-generation sequencing (NGS) analysis; species having abundance lower than 0.5% were indicated as “Others”. (**B**) The main Cyanobacteria species identified by microscopy observations: *Phormidium* sp. ETS-05, *Spirulina* sp. ETS-09, *Leptolyngbyaceae* sp. ETS-13 and *Oscillatoriaceae* sp. ETS-14. (**C**) Heatmap showing the relative abundance of main Cyanobacteria taxa identified from light microscopy observations and calculated from 16S rRNA amplicon sequencing. From left to right, results are represented as a function of increasing temperatures. Samples collected from maturation systems provided with protective roofs are indicated with *.

The NGS analyses of the phylum Cyanobacteria highlighted the presence of 12 main OTUs (Figure 5A). 05 was the most widespread species, with an average abundance of 41.4%. This species was detected in a significant amount in all mud samples at temperatures up to 45.7 °C (average of 52.6%). Interestingly, in tank-maturation systems, besides the presence of ETS-05, we observed the prevalence of another filamentous Cyanobacteria, member of *Oscillatoriaceae*, namely *Oscillatoriaceae* sp. ETS-14. This species showed an average relative abundance of 60.6% (of total Cyanobacteria) in tanks, compared to 1% detected in samples collected from ponds. At the opposite, maturation ponds in the central temperature range showed the presence of the OTU *Microcoleaceae* sp. ETS-17 (17.5% on average). Some OTUs were observed in relevant amounts only in individual cases, as *Chroococcales* sp. ETS-16 and *Cyanobacterium aponinum* ETS-03 (Table S2). At temperatures over 45.7 °C we observed clear changes in Cyanobacteria population composition, with a significant increase of the abundance of *Spirulina* spp. (ETS-09 and ETS-15) and *Leptolyngbyaceae* sp. ETS-13. The genus *Spirulina* was observed in all samples analyzed, however only from 45.7 °C it was detected in high abundance, with maximum values at 49.5 °C (21.1 and 35.8% for ETS-09 and ETS-15, respectively). *Leptolyngbyaceae* sp. ETS-13 was the dominant species at 54.7 °C, representing more than 80% of the Cyanobacteria population. Furthermore, at the same temperature, another two species assigned to *Synechococcaceae* were observed in higher abundance than in any other sample.

Results showed that the central temperature range (39.1–45.7 °C) of mud maturation leads to the highest Cyanobacteria content, biodiversity and community stability. Moreover, *Phormidium* sp. ETS-The biodiversity of Cyanobacteria at different conditions was studied also through microscopy analyses. These allowed cross check assignments based on NGS data analyses for the most abundant filamentous Cyanobacteria species (Figure 5B) and to carry out a wider investigation on all the mud samples collected in 2017. When morphological features were non-distinctive for the determination at species level, the main Cyanobacteria strains were assigned to genera or families [37,38,39,40,41], as for *Spirulina* spp. and *Leptolyngbyaceae* spp., while coccoid forms were merged in a unique group. Results, reported in the heatmap (Figure 5C), confirmed the indications provided by NGS data analysis. *Phormidium* sp. ETS-05 was the most widespread species identified by microscopy analyses in muds matured at temperatures up to 47.0 °C and showing, in most cases (20 spas), abundances higher than 50%. Only two samples (19b and 3a) collected at temperatures up to 47.0 °C showed lower abundance of ETS-05, with the main species represented by ETS-14 (Figure 5B), again in agreement with NGS data analyses. Results confirmed that the genus *Spirulina* was characterized by a medium-high temperature optimum, with an abundance steadily growing from 41.7 °C and up to 49.5 °C. Over 49.5 °C the composition of Cyanobacterial populations changed consistently; *Leptolyngbyaceae* spp. (ETS-13 as the main representative, Figure 5B) became the dominant family in mud samples at high temperatures, particularly in Spas 9, 21 and 16. Only in Spa 14 was the presence of 22% of coccoid species observed, probably including the species *Synechococcus* sp. ETS-19. In general, coccoids identified during microscopy observations appeared rather distributed on muds at different temperatures, confirming the presence of multiple species with different characteristics. In general, *Phormidium* sp. ETS-05 and *Leptolyngbyaceae* spp. were regarded as the main Cyanobacteria found in mud samples, and their abundance was significantly correlated with temperature. *Phormidium* sp. ETS-05 showed a moderate inverse correlation with temperature (Pearson coefficient *r* = −0.5926, *p*-value = 0.0023), while *Leptolyngbyaceae* spp. were directly correlated (*r* = 0.6242, *p* = 0.0011). Cyanobacteria abundances resulting from NGS analysis showed a good correlation with the Chl *a* content. In 3 samples, the Chl *a* values found in mature muds were lower (12.6, 15.5, 18.7 µg/g_MUD_ respectively) than the overall average (20.9 µg/g_MUD_). This was confirmed by the lowest relative abundances found for the Cyanobacteria phylum among all samples analyzed (respectively, 3.07, 6.97 and 3.83%). All spas characterized by a very high Cyanobacteria relative abundance showed also Chl *a* values higher than the average and were provided by protective roofs (Figure S7A,B). The only exception was represented by the sample a collected from Spa 14, characterized by high maturation temperature and by the exposure to high solar radiation. For this sample, the lowest Chl *a* content was observed (9.89 µg/g_MUD_), but the relative abundance of Cyanobacteria based on NGS analysis was higher than expected (17.7%). This sample presented a thick biofilm, composed of more than 80% from *Leptolyngbyaceae* sp. ETS-13 and characterized by a yellow color (Figure S7C,D), probably due to the high light exposure, that caused light stress in Cyanobacteria and, consequently, a decreased Chl *a* content. The most abundant filamentous species (ETS-05, ETS-14 and ETS-13) were also characterized in terms of cellular size, on the basis of measurements of trichome width and cellular length (*n* = 30), performed using high-magnification images acquired at the microscope. Trichomes of *Phormidium* sp. ETS-05 ranged from 4.0 to 7.4 µm (average 5.5 µm, SD 0.9 µm), with cell length from 3.0 to 8.8 µm (5.1 ± 1.1 µm) (Figure S7). The average values are consistent with those reported in literature for this species [23]. Species ETS-14 was characterized by trichomes measuring from 1.9 to 3.6 µm of width (2.9 ± 0.5 µm) and cell length of 1.5-3.8 µm (2.3 ± 0.6 µm). Trichomes of *Leptolyngbyaceae* sp. ETS-13 were 0.9–1.2 µm wide (1.1 ± 0.1 µm) and cells 2.8–4.9 µm long (3.8 ± 0.9 µm).

### 3.5. Phormidium sp. ETS-05 Genome Sequence

**Figure 6:**
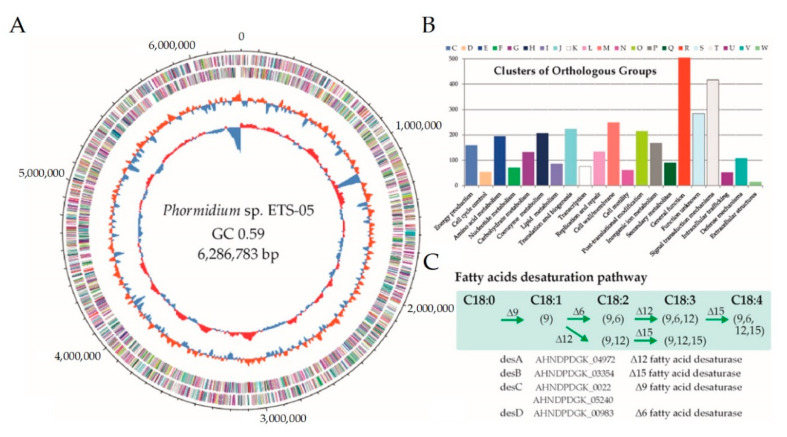
(**A**) Circular view of *Phormidium* sp. ETS-05 genome, showing from outside inward: (i) predicted coding sequences (CDSs) localized on forward/reverse strands and coloured according to the clusters of orthologous groups (COG), (ii) GC content (%), and (iii) GC skew. (**B**) Histogram reporting the number of genes assigned to each COG category. (**C**) Schematic representation of the manually-curated fatty acids pathway reporting the genes identified from the genome annotation.

Since *Phormidium* sp. ETS-05 is one of the crucial species involved in the mud maturation process, an accurate reconstruction of its genome sequence was obtained by assembling 180,370 long Nanopore reads (accounting for 0.73 Gbp in total) and 4.3 million paired-end Illumina reads (1.29 Gbp). The closed circular genome is composed of a single chromosome of 6,286,783 bp having a 59.09% GC content (Figure 6A).

Knowledge of the consensus ORI sequence for Cyanobacteria allowed a precise positioning of the starting point to be defined, while comparison with strictly related genomes allowed the proper assignment of forward and reverse strands. The gene finding and annotation process predicted a total of 5535 coding sequences (CDSs), 60 of which were identified as RNA genes, including 54 transfer RNAs (tRNAs), and 6 ribosomal RNA (rRNA) genes (Table 1). The GC content is quite similar across the entire genome sequence, but a clear peak around 1.13–1.20 Mbp is suggestive of a lateral gene transfer event. This large region including approximately 80 genes is characterized by the presence of numerous transposases and a type II toxin-antitoxin (TA) system.

Starting from 1471 Cyanobacterial genomes, a first phylogenetic analysis was performed using taxonomic-informative proteins. Then, on a subset of 76 selected species, average nucleotide identity was calculated allowing the identification of those strictly related to *Phormidium* sp. ETS-05 (Figure S7). Results revealed that the closest genome publicly available is *Oscillatoria acuminata* PCC 6304 [64], obtained from the Gomond herbarium. The size of *Phormidium* sp. ETS-05 genome is 18% smaller in comparison with that of *O. acuminata* (7.69 Mbp), however, the gene content is comparable, being 5535 and 5953 respectively, and evidencing a higher gene density in ETS-05. Genomic functional annotation was obtained by assigning the predicted genes to the COG categories (Figure 6B) and to the functionally related protein families (FIGfams) of the SEED (Simple Exploration of Ecological Data) subsystem (Table 1). Regarding COG analyses, more than 400 genes were assigned to the “Signal transduction mechanisms” category, suggesting that *Phormidium* sp. ETS-05 possess sophisticated mechanisms of response to the environmental stimuli. Other highly represented functional categories are involved in “post-translational modification” and in “cell-wall/membrane” organization. In order to have a high-resolution level of the functional properties, the predicted proteins were assigned to the KEGG metabolic pathways. According to this characterization, 55 KEGG modules were identified as “complete”, including three related to carbon fixation: the Calvin cycle, the conversion of ribulose-5P into glyceraldehyde-3P and the reverse mechanism of conversion. Additionally, all genes belonging to the photosystems I and II and to the anoxygenic photosystem II were found in the genome. According to KEGG results, *Phormidium* sp. ETS-05 is potentially able to perform both “assimilatory nitrate reduction” (from nitrate to ammonia) and “assimilatory sulfate reduction” (from sulfate to H2S). Finally, manual inspection of lipid metabolism revealed that the *Phormidium* sp. ETS-05 genome encodes a complete set of genes for lipid metabolism (Figure 6C).

## 4. Discussion

The analysis of physico-chemical parameters of the Euganean District thermal waters revealed a variability among the studied sampling sites. This was expected given that each spa carries out the mud maturation independently, utilizing the same virgin clay but their own mud maturation systems and thermal waters flowing from different wells widespread in the territory. The variability observed in pH and EC values was comparable with those reported by Pola et al., 2015 and Calderan et al., 2020 [5,7], and depends mainly on the location of the wells from which thermal waters are extracted by spas to feed mud maturation systems. As regards temperature, differences can be explained by analyzing the functioning of maturation systems and by interviewing spa employers who indicate the difficulty of maintaining a constant water temperature, particularly in ponds. In fact, in this traditional maturation system, several ponds are directly connected together (Figure S1) and their water temperature is controlled using empirical methods, such as manually reducing or augmenting the flow rate and the depth of thermal water covering the muds. Thus, working on one pond of the plant means causing temperature variations also in the nearby ones, leading to temporary deviations from the desirable mud maturation temperature. The control of this parameter in tank maturation systems is also made by regulating the water flow rate, but this operation is done independently for each tank as they are not interconnected (Figure S1), making it easier to maintain a desired water temperature. In this work, Chl *a* was confirmed to be a reliable indicator of Cyanobacteria colonization, as previously reported [14,65,66], at least for mud maturation temperatures up to 47 °C, beyond which Cyanobacteria abundance and species composition change, making the correlation no more direct. Values found in mature muds were similar to those previously obtained for the surface layer of muds from maturation ponds of Euganean Spas [15], and higher than values found by Calderan et al., 2020 [7], which considered the mixed mature muds ready for therapies. Our findings showed that, among water parameters, temperature control is crucial to increase the final Chl *a* content of mature muds. The range from 37 to 47.0 °C allowed us to obtain the highest Chl *a* levels, with values even higher if maturation ponds or tanks were provided with shading roofs protecting the Cyanobacteria growing on muds from light stress. The influence of operating parameters was evaluated also on the total amount and biodiversity of microbial communities colonizing mature muds, paying attention to Cyanobacteria populations. In the literature, only few studies are focused on the description of microorganisms colonizing peloids used for therapeutic purposes [2]. Most of them are limited to the characterization of only one or a small number of species, mainly using microscopy techniques [12,67,68,69,70] or culture-dependent approaches [71,72,73]. As regards the Euganean area, a survey of photosynthetic microorganisms based on microscopy observations dating back to 1975 [25]. Furthermore, only polyphasic characterization were conducted on the isolated species *Phormidium* sp. ETS-05 [23], *Cyanobacterium aponinum* ETS-03 [74] and *Themoleptolyngbya albertanoe* ETS-08 [27,75]. However, the biodiversity of the microbiota colonizing Euganean muds is much more complex. Considering that the maturation is a semi-natural process, and that much more data on the microbiota colonizing the natural springs are available, results of our work are also compared with data collected for natural thermal or hot springs around the world. Despite our study being conducted on a high number of samples showing a wide temperature range (33.8–54.7 °C), sampling sites analyzed revealed similar phyla profiles, with Cyanobacteria and Chloroflexi as dominant phyla in almost all samples, as observed in thermophilic mats from natural hot springs of Yellowstone [76], Western [77] and Northern Thailand [78]. The bacterial phyla Proteobacteria and Bacteroidetes were also abundant in Euganean thermal muds, as in many other natural thermal springs [29,79]. The taxa composition of the main phyla Chloroflexi, Proteobacteria and Bacteroidetes enabled identification of 13 genera and 14 species, mainly belonging to Proteobacteria. Among them, the most abundant genera and species were found and isolated from thermal and hot springs around the world [80,81,82,83,84,85,86] and growing at temperatures and pH ranges including those found in mud maturation systems of Euganean spas. No known pathogenic species were found among the most abundant found in the Euganean muds, as observed by Baldovin et al. 2020 [87], which investigated the thermal mud’s hygienic quality, checking for the absence of specific microorganisms using classic microbiological methods. The global taxa composition was stable and statistical analysis was performed considering all samples showing no significant variations on microbial biodiversity. However, sampling sites at the lowest and the highest temperatures showed Shannon indexes slightly lower than in other samples. Probably, the temperature range of Euganean muds is too narrow to determine a high selectivity on the species, as happens instead in environments with a wider range of temperatures, which show a decrease of biodiversity over 70 °C [78]. A deep analysis of Cyanobacteria community, performed considering both molecular and microscopy data, highlighted that Cyanobacteria population is stable in the central temperature range (37–47 °C), while showing marked changes at temperature higher than 47 °C. In general, the central range revealed the highest species biodiversity, with the target species *Phormidium* sp. ETS-05 representing in most cases more than half of the population. This is particularly evident in ponds, while in tanks the species *Oscillatoriaceae* sp. ETS-14 was preferentially found. Interestingly, both these *Oscillatoriaceae* showed high nucleotide identity on the 16S rRNA gene with strains found in Greek natural thermal springs at 41 °C. ETS-14 showed 100% identity with strain ThrGT9, and ETS-05 99.2% with strain ElfPHct20 [88]. In this range, among other species, one of those previously studied in great detail is *Cyanobacterium aponinum* ETS-03, found also in Blue Lagoon in Iceland, at temperatures from 34 to 42 °C [89]. *Leptolyngbyaceae* sp. ETS-13, *Spirulina* spp. ETS-15 and ETS-09 were identified in the present study, dominating the Cyanobacteria populations only over 45 °C. The species ETS-14 showed 100% identity with the strains ST7T_1CY_22/23, found in natural Meskoutine spring in Algeria at 60 °C, [36]. *Spirulina* sp. ETS-09 was similar (99.6% of identity) to strain ElfSPR27 found in Greek natural thermal springs at 41 °C [88], while species ETS-15 was found in “Snake Pit” hot spring (50 °C) at Yellowstone National Park, USA (100% identity with strain CCC Snake P. Y-85, [90]). At 54.7 °C a relevant abundance of *Synechococcus* sp. ETS-19 was recovered and this species showed an identity of 100% with strain C9 found at Octopus Spring (50 °C) in Yellowstone [91]. Although many species observed in mature muds show high identities with species found in natural thermal or hot springs around the world, Euganean thermal muds showed a unique and specific Cyanobacteria composition. Molecular and microscopy analysis correlate well with Chl *a* mud content, revealing that temperature control is fundamental to ensure a correct proceeding of mud maturation process. The range 37–47 °C resulted in the highest Chl *a* values in the mud. This goal is clearly favored by the stability in the Cyanobacteria population and by the presence of the target species *Phormidium* sp. ETS-05. Our work also identified species never observed before in the Euganean Thermal District, such as *Leptolyngbyaceae* sp. ETS-13, *Spirulina* sp. ETS-15 and *Synechococcus* sp. ETS-19 and highlighted also that some of the previously isolated strains, such as *Cyanobacterium aponinum* ETS-03 and *Thermoleptolyngbya albertanoe* ETS-08, are detected only at low abundances. Moreover, the strains *Leptolyngbyaceae* sp. ETS-13 and *Spirulina* sp. ETS-09 have been recently isolated and will be the object of a polyphasic characterization. Experiments will be conducted to verify if these species are able to produce some interesting high-value molecules which can contribute to the final therapeutic effects of muds treatments. The analysis of the target species *Phormidium* sp. ETS-05 16S rRNA gene sequence revealed that the closest species are *Phormidium* sp. ElfPHct20 from Greek natural thermal springs [88], with 98.26% of identity and *Koinonema pervagatum* 63PC at 98.04% of identity [92]. In the work of Buch and co-workers based on the comparison of *Phormidium* sp. ETS-05 [23] and *Phormidium* sp. ElfPHct20 [88] 16S rRNA gene sequences, all these strains were proposed to be conspecific. However, in our opinion, further investigations, such as a direct comparison of morphology of the different strains under different conditions, as well as their polyphasic characterization and genome sequencing, are needed to confirm this hypothesis. Our micrographs of the strain *Phormidium* sp. ETS-05 (Figure S8) in fact, clearly showed the presence of a sheath surrounding trichomes, while this taxonomic character is reported to be absent in the description of the species *Koinonema pervagatum* [92]. Furthermore, culturing *Phormidium* sp. ETS-05 at 40 °C in BG11 medium we measured cell sizes up to 8.7 µm, larger than those reported for *K. pervagatum* (Figure S8). These findings support the idea that molecular markers 16S-23S ITS are not always conclusive to define species [41,93]. The high-quality genome sequence of *Phormidium* sp. ETS-05 obtained with a combined strategy based on Illumina and Nanopore reads revealed with high level of detail the complete set of genes encoded. The reconstruction of the complete genome is extremely important in order to uncover also the genes located in the genomic regions more difficult to assemble such as those including perfect and imperfect repeats. This allowed reconstructing the complete picture of the metabolic potential and it will be also important in future studies to determine variation in the expression of genes encoding enzymes involved in specific molecules biosynthesis, included in the European Patent (EP1571203). In fact, the recovery of the complete genome is particularly important not only for a detailed characterization of the encoded functional pathways, but also to serve as a reference for future studies. The galactolipids mono- and the digalactosyldiacylglycerol (MGDG and DGDG) produced by the target species *Phormidium* sp. ETS-05 were identified in previous works as molecules with anti-inflammatory effects [16,18,19,21]. Furthermore, the ability of *Phormidium* sp. ETS-05 to produce other molecules such as exopolysaccharides (EPS) with anti-inflammatory effects was recently studied [22]. Knowledge of genes involved in the pathways of the synthesis of these molecules will be pivotal in understanding how to promote, change the culture conditions of, and produce metabolites important for the therapeutic effects of Euganean muds.

## 5. Conclusions

This work provides, for the first time, an in-depth study conducted using a combination of molecular and microscopy techniques to evaluate the microbiota composition of the mature muds collected from Euganean spas. Results revealed a stable microbial community, showing a general phyla composition comparable to those found in similar conditions in natural springs around the world. The molecular analysis of Cyanobacteria colonizing muds revealed the presence of species already reported in this environment, but also species not previously identified. The comparison with other thermal sites highlighted that the Cyanobacteria composition of Euganean muds is peculiar and unique, but more comprehensive studies performed with homogenous strategies will in the future allow us to gain a more clear understanding of the microbial composition in diverse locations. Differences noted among samples were determined by environmental and operating conditions set during the maturation process. In particular, temperature is the most relevant, leading to the highest Chl *a* content, a stable Cyanobacteria population composition and high abundance of the target species *Phormidium* sp. ETS-05 when maintained in the range 37–47 °C. At lower and higher temperatures, populations lose their stability, showing a significant change in species composition, a decreased biodiversity and a lower Cyanobacteria abundance. For these reasons, keeping the temperature in the range 37–47 °C and providing maturation systems of protecting roofs are suggested as the new standards for the mud maturation process. The genome sequence of *Phormidium* sp. ETS-05 will be the base for future studies to understand how operating parameters influence the production of active therapeutic molecules (lipids and polysaccharides). In particular, the production of exopolysaccharides by pure cultures of *Phormidium* sp. ETS-05, and the corresponding anti-inflammatory and antioxidant effects, suggests an interesting target for further studies, including the activity test of EPS extracted directly from the mature muds. This work, besides the improvement of scientific data on such a peculiar thermal site, provides an important contribution to knowledge in the field of peloids and pelotherapy.

## Figures and Tables

**Table 1:** Genomic characteristics of *Phormidium* sp. ETS-05.

Taxonomy Based on ANI	Cyanobacteria; *Oscillatoriophycideae*; *Oscillatoriales*; *Oscillatoriaceae*; *Oscillatoria*
Best hit 16S rRNA nrNCBI (similarity %)	*Phormidium* sp. ElfPHct20 (98.26)
Genome size (Mbp)	6.29
GC content (%)	59.09
CDSs	5535
CDSs assigned to FIGfams	3140
NCBI BioProject	PRJNA622676

## Supplementary Materials

The following are available online at https://www.mdpi.com/2076-2607/8/10/1590/s1, Figure S1: Artificial ponds and tanks used by Spas for mud maturation process without (A) and with protective roofs (B). Representation of a typical pond system (C). Figure S2: Average rarefaction curves (n = 2) of each sampling site (A), sequencing data and diversity parameters reported with average and standard deviations of replicates for each sampling site (B). Figure S3: Taxonomic analysis performed considering the 1471 genomes assigned to the Cyanobacteria and deposited in the NCBI microbial genome database (A). The average nucleotide identity calculated on the entire genome sequence, and represented here as a tree, shows the relationships among 76 selected Cyanobacteria species including *Phormidium* sp. ETS-05 (B). Figure S4: Heatmap representing the relative abundances of the OTUs assigned to Chloroflexi and having relative abundance higher than 0.5% across different temperatures and sampling sites. Figure S5: Heatmap representing the relative abundances of the OTUs assigned to the Proteobacteria and having abundance >0.5% on total phyla. Figure S6: Heatmap reporting the relative abundances of the main OTUs (abundance >0.5% on total phyla) of Bacteroidetes at different temperatures and sampling sites. Figure S7: Green biofilms growing on the muds surface in maturation systems provided with protective roofs (A,B), and yellow biofilms from ponds or tanks exposed to solar radiation (C,D). Figure S8: Micrographs of *Phormidium* sp. ETS-05 cultured at 40 °C in BG11 medium. Table S1: Average values of physico-chemical parameters (n = 4) of water samples from thermal Spas in Abano, Montegrotto and Battaglia Terme in 2017 and 2018. Table S2: 16S rRNA sequences of OTUs corresponding to the most abundant taxa.
