# Supplementary material for: Microbiota of the Therapeutic Euganean Thermal Muds with a Focus on the Main Cyanobacteria Species
Source: Microorganisms. 2020 Oct 15;8(10):1590. doi: 10.3390/microorganisms8101590 (PMC7650686; doi:10.3390/microorganisms8101590)
Supplement: Supplementary file 1 [file microorganisms-08-01590-s001.zip › microorganisms-932556-supplementary-published (final)/microorganisms-932556-supplementary-proofed.docx]

**Microbiota of the therapeutic Euganean thermal muds and focus on the main cyanobacteria species**

Barbara Gris^1,3^, Laura Treu^1^, Raffaella Margherita Zampieri^1^, Fabrizio Caldara^2^, Chiara Romualdi^1^, Stefano Campanaro^1,4^, Nicoletta La Rocca*^1,3^

1 Department of Biology, University of Padova, Via U. Bassi 58/B, 35121, Padova, Italy

2 Pietro d’Abano Thermal Studies Center, Via Jappelli 5, 35031, Abano Terme, Italy

3 Botanical Garden, University of Padova, Via Orto Botanico 15, 35123, Padova, Italy

4 CRIBI Biotechnology Center, University of Padova, 35121 Padova, Italy

Corresponding author: Associate Prof. Nicoletta La Rocca

Department of Biology, University of Padova

Via U. Bassi 58/B, 35131, Padova, Italy

+390498276273

nicoletta.larocca@unipd.it

Microbiota of Euganean therapeutic thermal muds

**Table S1:** Average values of physico-chemical parameters (n=4) of water samples from thermal Spas in Abano, Montegrotto and Battaglia Terme in 2017 and 2018. Numbers (1-29) identifies the Spas, letters (a,b,c,d) indicate different samplings sites or times from the same Spas. P and T distinguish maturation systems adopted as “pond” or “tank”, respectively. Protective roof indicates the presence of structures that protect tanks or ponds from solar radiation. Sampling selected for molecular analysis are highlighted in grey. Minimum and maximum values are indicated in blue and red, respectively. (Abbreviations: electric conductivity (EC), total dissolved solids (TDS), Chlorophyll *a* (Chl *a*)).

| Sampling site ID | Spa Location | Sampling Year | Temperature (°C) | pH | EC (mS/cm) | TDS (ppm) | Protective roof | Chl *a* (µg/g_MUD_) |
| --- | --- | --- | --- | --- | --- | --- | --- | --- |
| 1a-P | Montegrotto | 2017 | 41.7 | 7.68 | 7.56 | 3810 | YES | 28.3 |
| 1b-P | Montegrotto | 2018 | 36.9 | 7.71 | 7.54 | 3790 | YES | 18.0 |
| 2a-T | Abano | 2017 | 42.6 | 7.24 | 9.03 | 4500 | YES | 56.2 |
| 2b-T | Abano | 2018 | 46.2 | 7.31 | 9.09 | 4540 | NO | 11.6 |
| 3a-P | Abano | 2017 | 41.7 | 7.68 | 7.89 | 3930 | NO | 12.5 |
| 3b-P | Abano | 2018 | 51.6 | 7.73 | 8.35 | 4090 | NO | 14.2 |
| 4a-T | Abano | 2017 | 47.0 | 7.35 | 7.02 | 3540 | YES | 42.6 |
| 4b-T | Abano | 2018 | 41.4 | 7.43 | 6.98 | 3490 | YES | 23.2 |
| 5a-P | Abano | 2017 | 41.8 | 7.04 | 8.88 | 4440 | NO | 22.4 |
| 5b-P | Abano | 2018 | 27.4 | 7.55 | 8.70 | 4390 | NO | 12.0 |
| 6a-T | Abano | 2018 | **60.3** | 6.83 | 9.50 | 4750 | NO | 9.26 |
| 7a-P | Abano | 2017 | 33.8 | 7.49 | 9.78 | 4890 | NO | 12.6 |
| 7b-P | Abano | 2018 | 47.1 | 7.52 | 9.81 | 4880 | NO | 11.6 |
| 8a-P | Abano | 2018 | 43.0 | 7.81 | **9.99** | **5000** | NO | 11.5 |
| 9a-P | Abano | 2017 | 52.6 | 7.46 | 8.76 | 4380 | NO | 20.3 |
| 9b-P | Abano | 2018 | 44.6 | 7.65 | 8.59 | 4260 | NO | 20.6 |
| 10a-T | Abano | 2017 | 39.6 | **6.75** | 8.43 | 4200 | NO | 21.8 |
| 11a-P | Abano | 2017 | 45.4 | 7.48 | 9.54 | 4800 | NO | 24.8 |
| 11b-P | Abano | 2018 | 41.5 | 7.67 | 9.48 | 4780 | NO | 21.2 |
| 12a-P | Montegrotto | 2017 | 44.8 | 7.76 | 8.55 | 4230 | NO | 20.3 |
| 13a-P | Montegrotto | 2018 | 43.8 | 7.76 | 8.10 | 4060 | NO | 10.2 |
| 14a-P | Montegrotto | 2017 | 54.7 | 7.47 | 7.14 | 3570 | NO | 9.89 |
| 15a-P | Montegrotto | 2017 | 38.7 | **8.06** | 8.88 | 4470 | NO | 16.8 |
| 15b-P | Montegrotto | 2018 | 42.9 | 7.89 | 8.99 | 4500 | NO | 12.3 |
| 16a-P | Montegrotto | 2017 | **21.5** | 7.57 | 9.54 | 4770 | NO | 8.93 |
| 16b-P | Montegrotto | 2017 | 55.2 | 7.59 | 9.24 | 4590 | NO | 11.4 |
| 16c-P | Montegrotto | 2018 | 22.5 | 7.94 | 9.47 | 4690 | NO | 10.8 |
| 16d-P | Montegrotto | 2018 | 36.2 | 7.69 | 9.33 | 4530 | NO | 12.6 |
| 17a-P | Montegrotto | 2017 | 40.6 | 7.66 | 8.73 | 4410 | NO | 15.5 |
| 18a-P | Montegrotto | 2017 | 43.4 | 7.78 | **4.47** | **2250** | NO | 18.2 |
| 18b-P | Montegrotto | 2018 | 29.3 | 7.67 | 4.75 | 2350 | NO | 14.0 |
| 18c-P | Montegrotto | 2018 | 42.3 | 7.73 | 4.89 | 2420 | YES | 25.2 |
| 19a-T | Montegrotto | 2017 | 39.1 | 7.31 | 9.63 | 4830 | YES | 56.5 |
| 19b-T | Montegrotto | 2017 | 39.8 | 7.23 | 9.60 | 4710 | YES | **75.7** |
| 19c-T | Montegrotto | 2018 | 39.9 | 7.33 | 9.74 | 4930 | NO | 19.9 |
| 20a-P | Abano | 2017 | 45.4 | 7.65 | 5.19 | 2550 | NO | 20.3 |
| 20b-P | Abano | 2018 | 56.7 | 7.69 | 5.22 | 2530 | NO | 17.2 |
| 21a-P | Abano | 2017 | 54.6 | 7.57 | 5.22 | 2640 | NO | 8.05 |
| 21b-P | Abano | 2018 | 49.3 | 7.64 | 5.47 | 2690 | NO | 8.13 |
| 22a-P | Abano | 2017 | 39.2 | 7.72 | 5.82 | 2970 | YES | 52.2 |
| 22b-P | Abano | 2018 | 50.0 | 7.85 | 5.86 | 2950 | NO | **6.62** |
| 23a-P | Abano | 2017 | 37.0 | 7.48 | 6.42 | 3210 | YES | 29.0 |
| 23b-P | Abano | 2017 | 49.5 | 7.25 | 6.90 | 3450 | YES | 18.7 |
| 23c-P | Abano | 2017 | 45.7 | 7.43 | 6.30 | 3150 | YES | 42.8 |
| 23d-P | Abano | 2018 | 45.3 | 7.40 | 6.49 | 3210 | NO | 13.1 |
| 24a-P | Abano | 2017 | 44.3 | 8.01 | 6.84 | 3420 | NO | 18.0 |
| 24b-P | Abano | 2017 | 40.5 | 7.94 | 6.85 | 3430 | NO | 10.1 |
| 25a-P | Montegrotto | 2018 | 43.3 | 7.87 | 6.87 | 3410 | NO | 14.7 |
| 26a-P | Montegrotto | 2017 | 40.9 | 7.95 | 6.33 | 3180 | YES | 36.2 |
| 26b-P | Montegrotto | 2018 | 40.7 | 7.90 | 6.23 | 3100 | NO | 14.9 |
| 27a-P | Abano | 2018 | 43.0 | 7.69 | 9.55 | 4790 | YES | 16.9 |
| 28a-P | Battaglia | 2017 | 45.9 | 7.26 | 3.66 | 1830 | NO | 24.5 |
| 29a-T | Abano | 2017 | 41.4 | 7.52 | 7.01 | 3490 | YES | 23.3 |


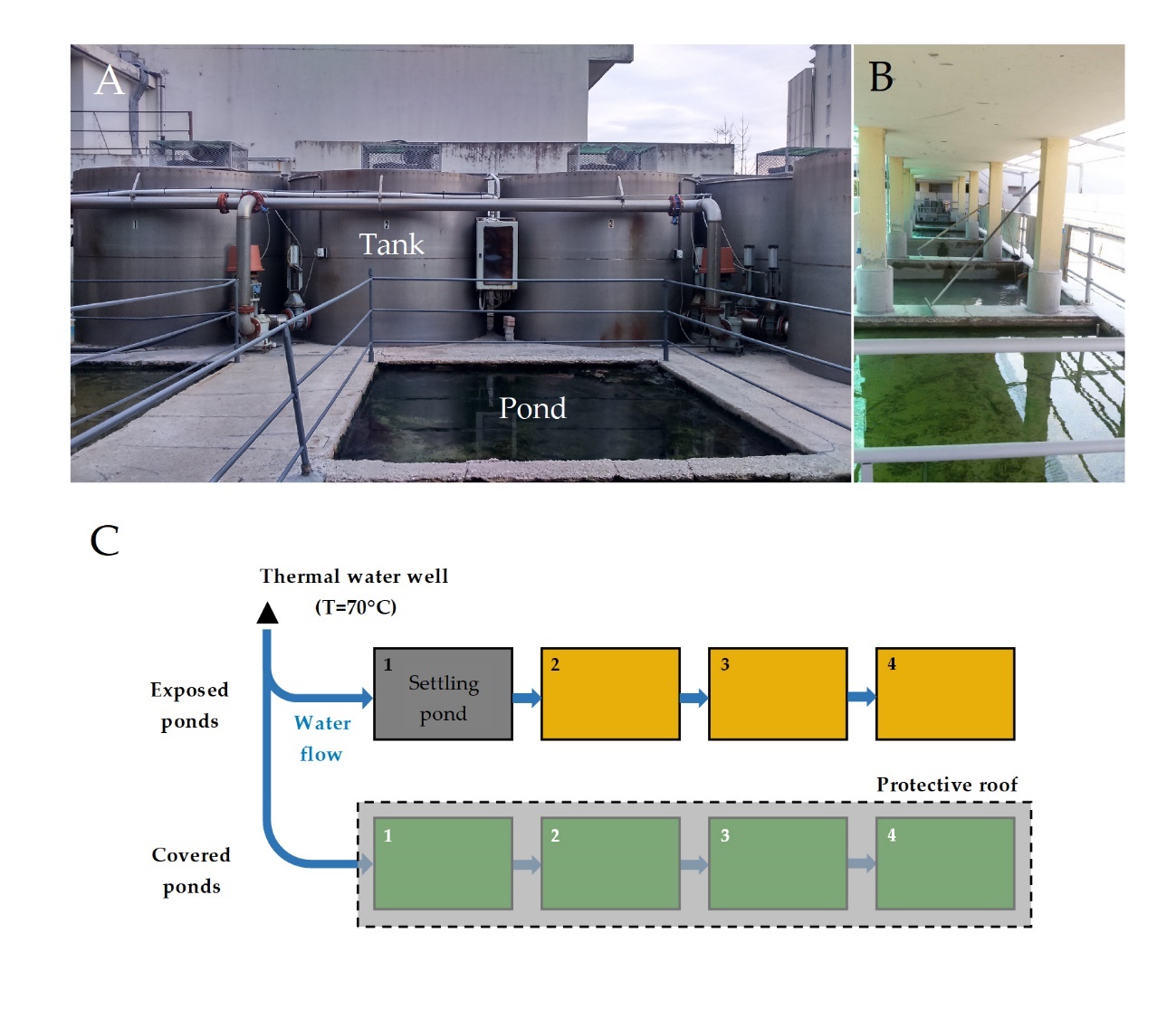


**Figure S1:** Artificial ponds and tanks used by Spas for mud maturation process without (A) and with protective roofs (B). Representation of a typical pond system (C).


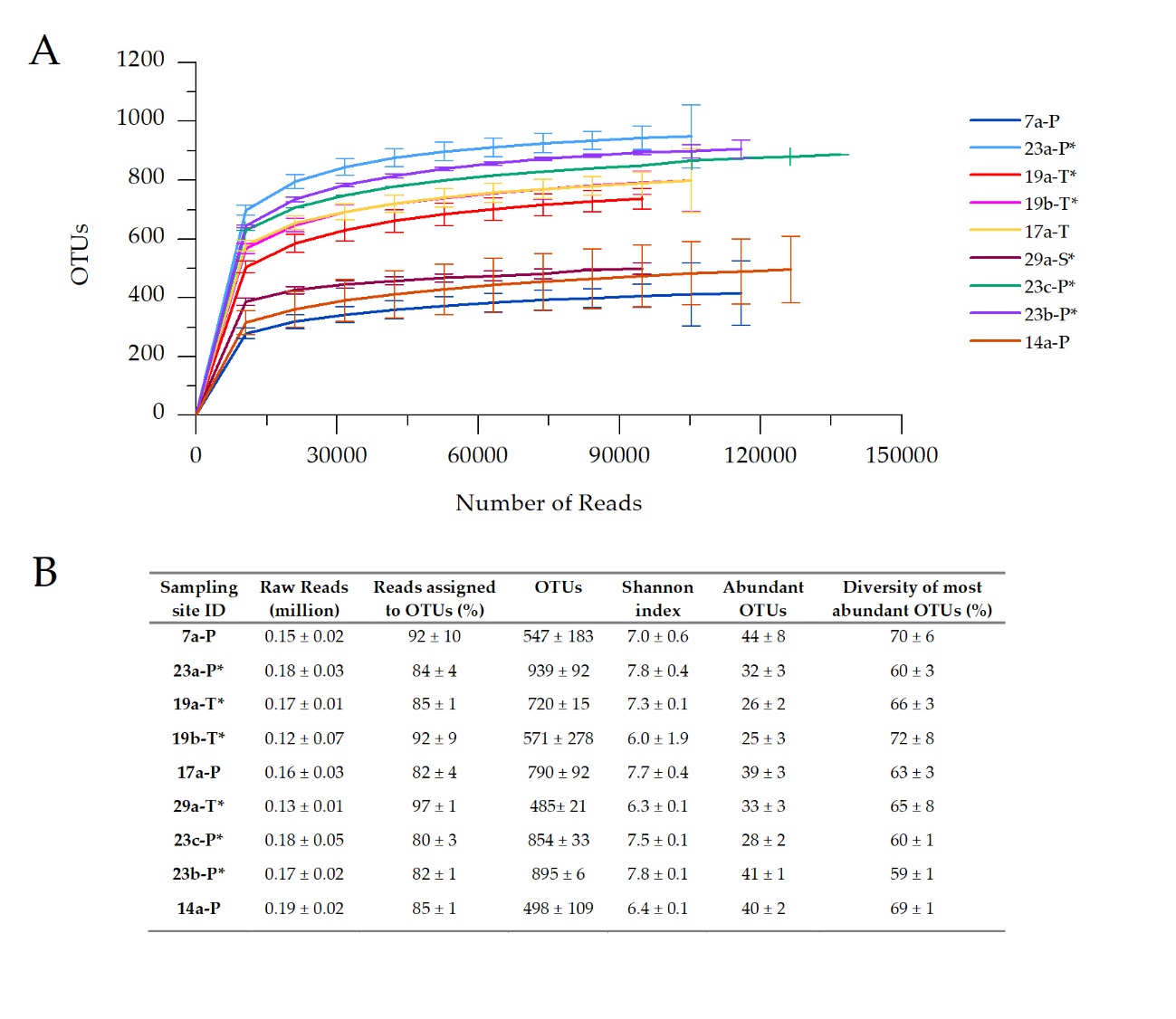


**Figure S2:** Average rarefaction curves (n=2) of each sampling site (A), sequencing data and diversity parameters reported with average and standard deviations of replicates for each sampling site (B). (Samples collected from maturation systems provided with protective roofs are indicated with *).


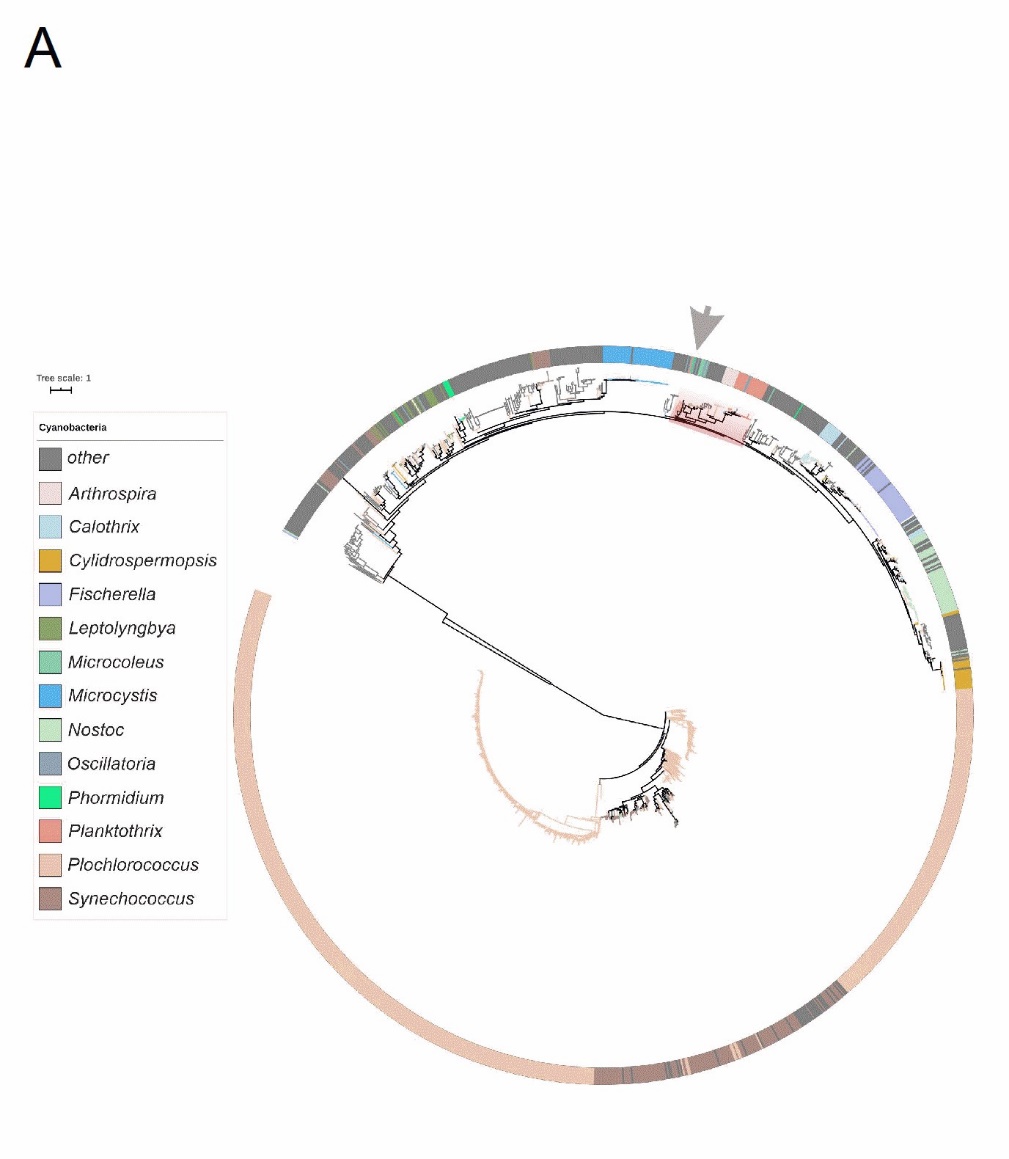


**
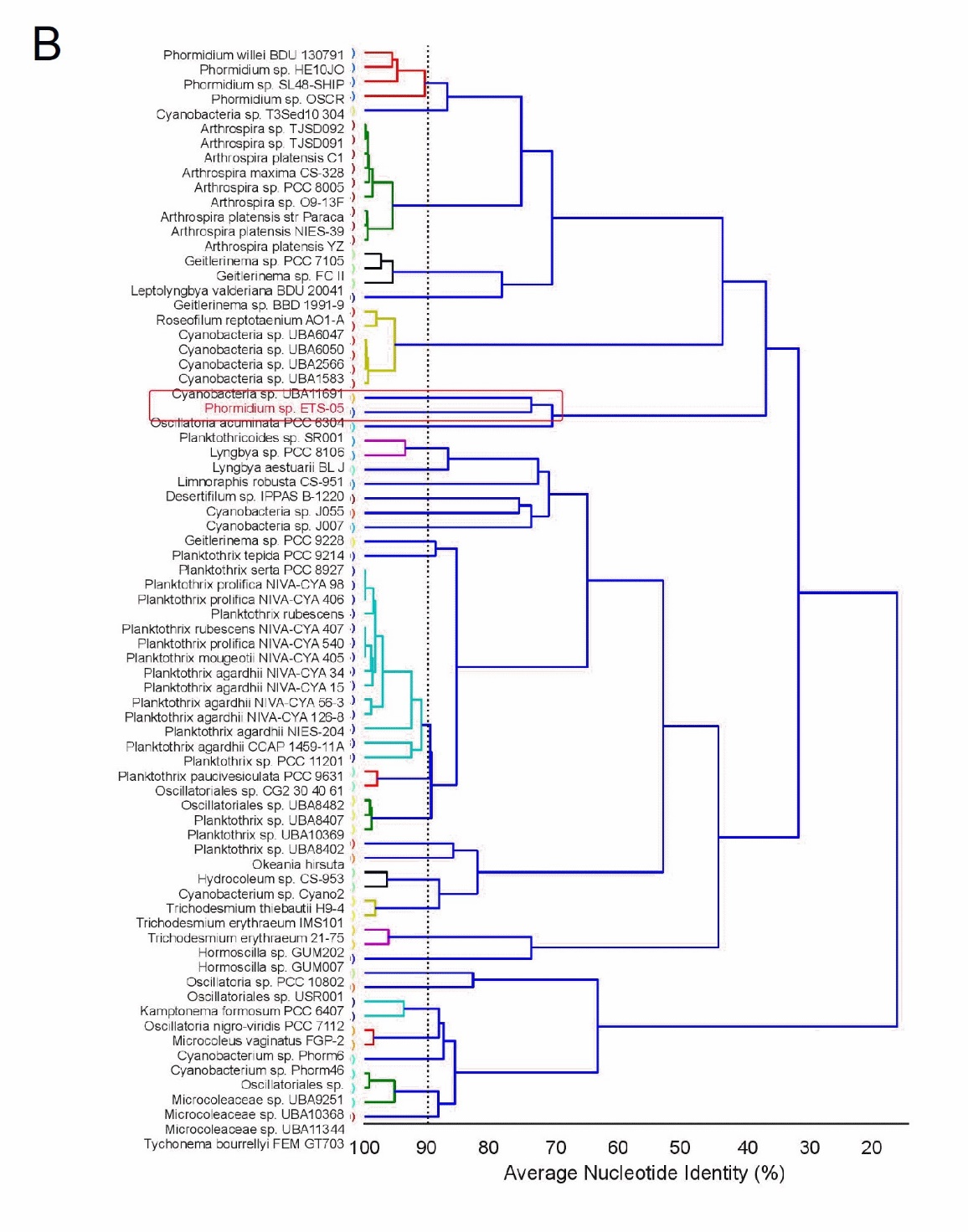
**

**Figure S3:** Taxonomic analysis performed considering the 1471 genomes assigned to the Cyanobacteria and deposited in the NCBI microbial genome database (A). Analysis was performed considering 400 taxonomic informative proteins present in the genomes (see materials and methods for details). Colors in the external ring are reported according to the assignment at genus level (not all the genera are reported) and unclassified species are marked in dark grey. The arrow indicates the position of the *Phormidium* sp. ETS-05 in the tree. The red box highlights the 76 species selected for the average nucleotide identity analysis. The average nucleotide identity calculated on the entire genome sequence, and represented here as a tree, shows the relationships among 76 selected Cyanobacteria species including *Phormidium* sp. ETS-05 (highlighted in red) (B). The dotted vertical line indicates the 90% identity threshold.


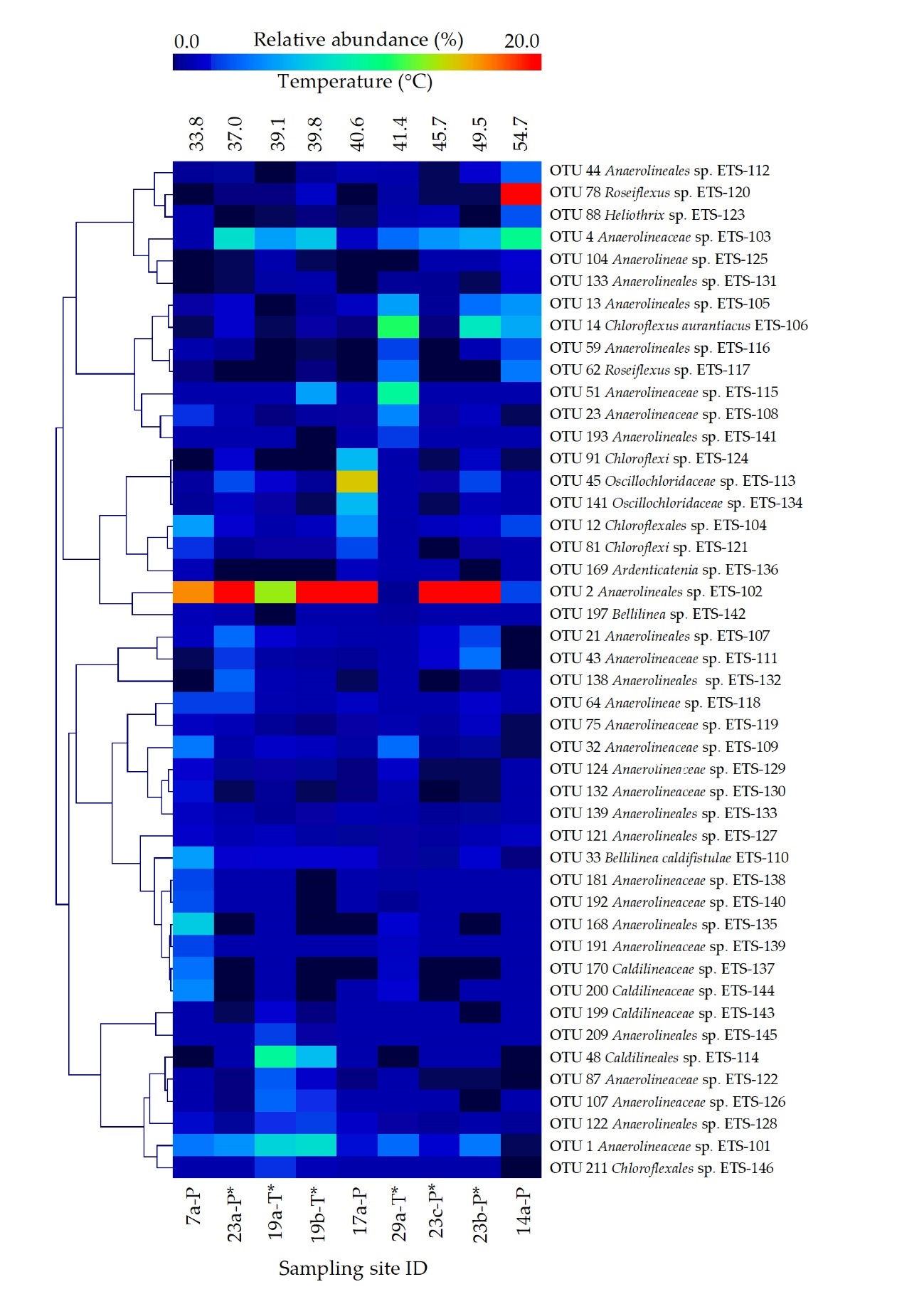


**Figure S4:** Heatmap representing the relative abundances of the OTUs assigned to Chloroflexi and having relative abundance higher than 0.5% across different temperatures and sampling sites. Samples collected from maturation systems provided with protective roofs are indicated with (*).


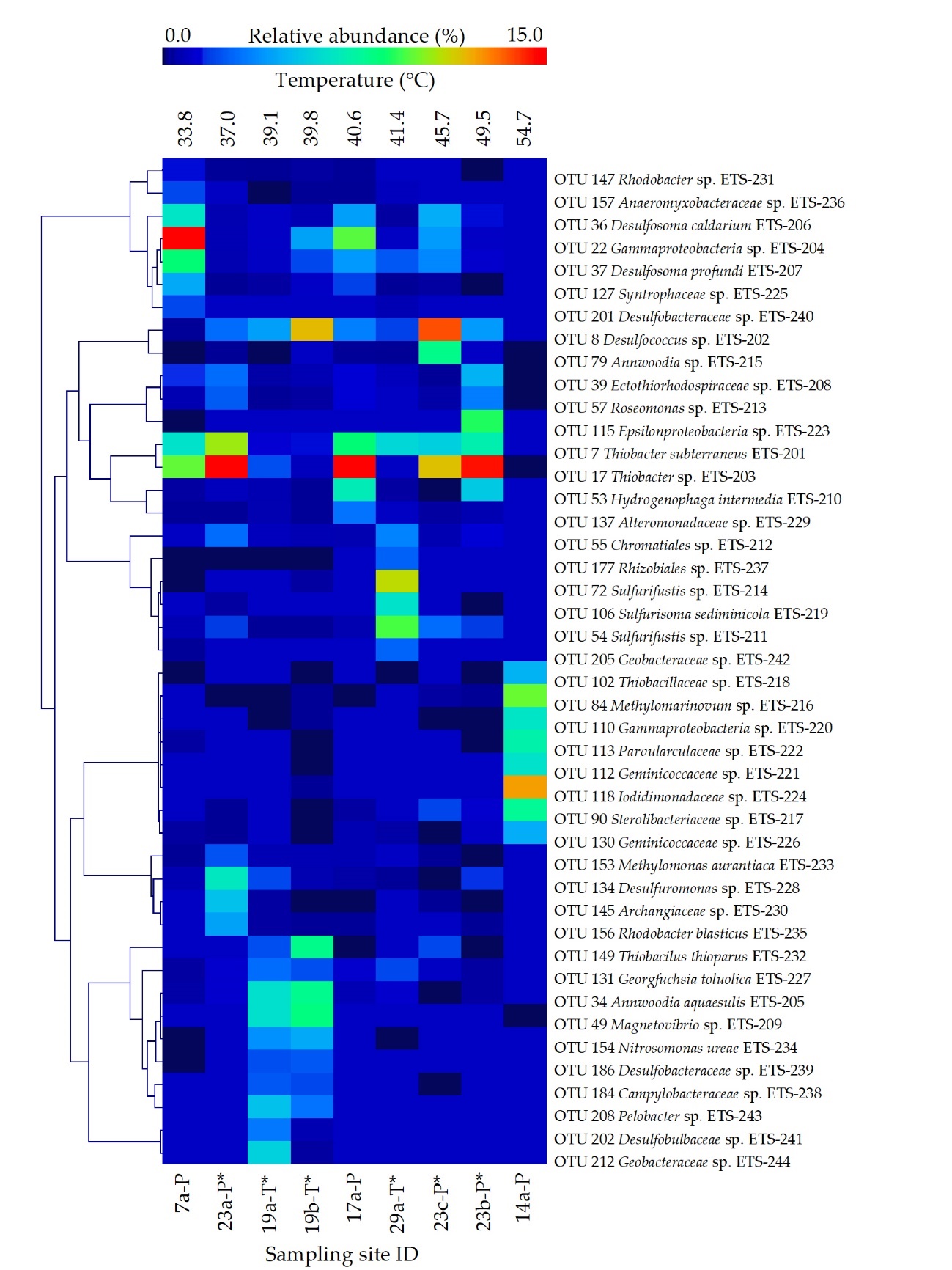


**Figure S5:** Heatmap representing the relative abundances of the OTUs assigned to the Proteobacteria and having abundance >0.5% on total phyla. Samples were obtained at different temperatures and sampling sites, and those collected from maturation systems provided with protective roofs are indicated with *.


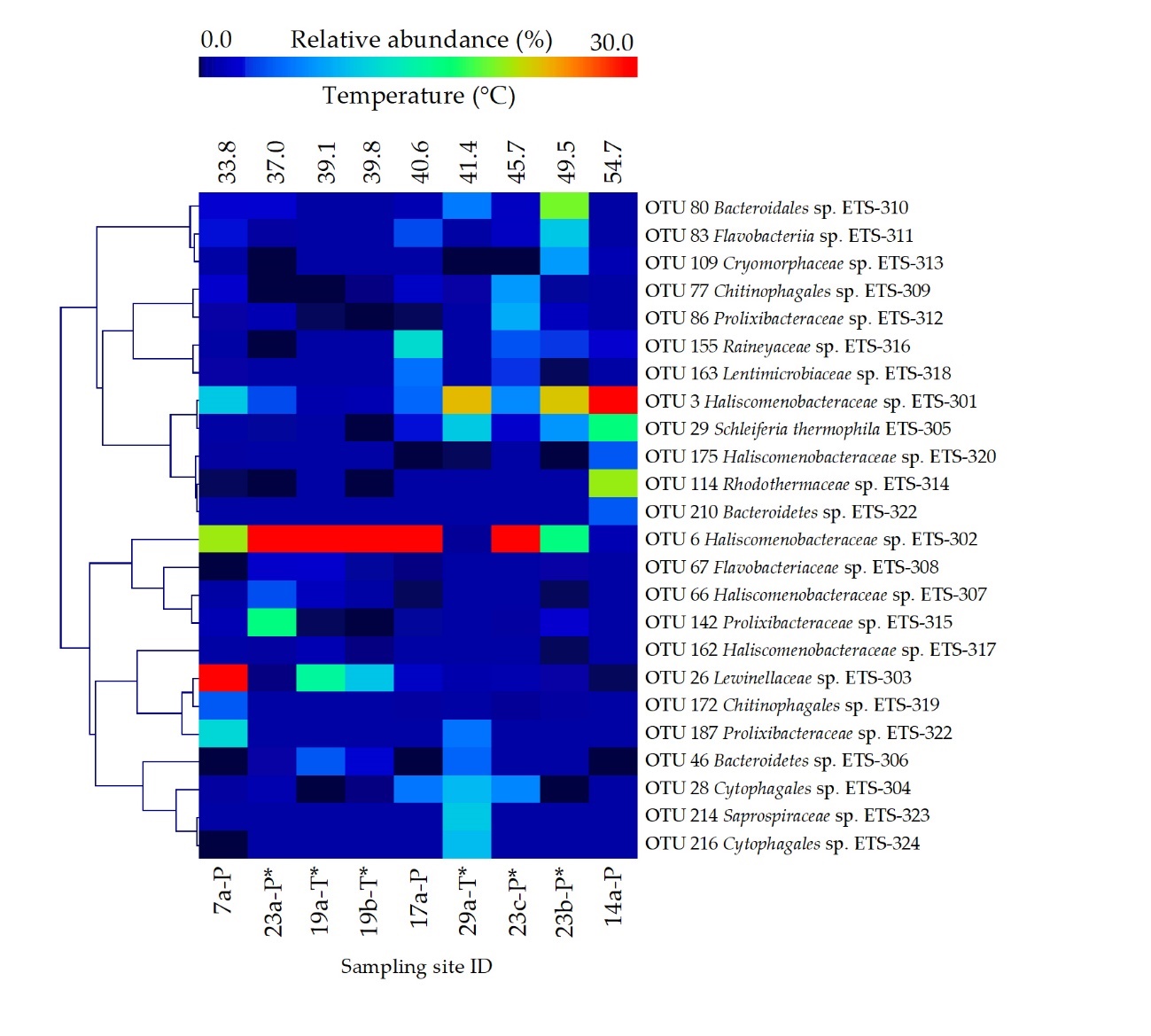


**Figure S6:** Heatmap reporting th**e** relative abundances of the main OTUs (abundance >0.5% on total phyla) of Bacteroidetes at different temperatures and sampling sites. Sample collected from maturation systems provided with protective roofs are indicated with *.


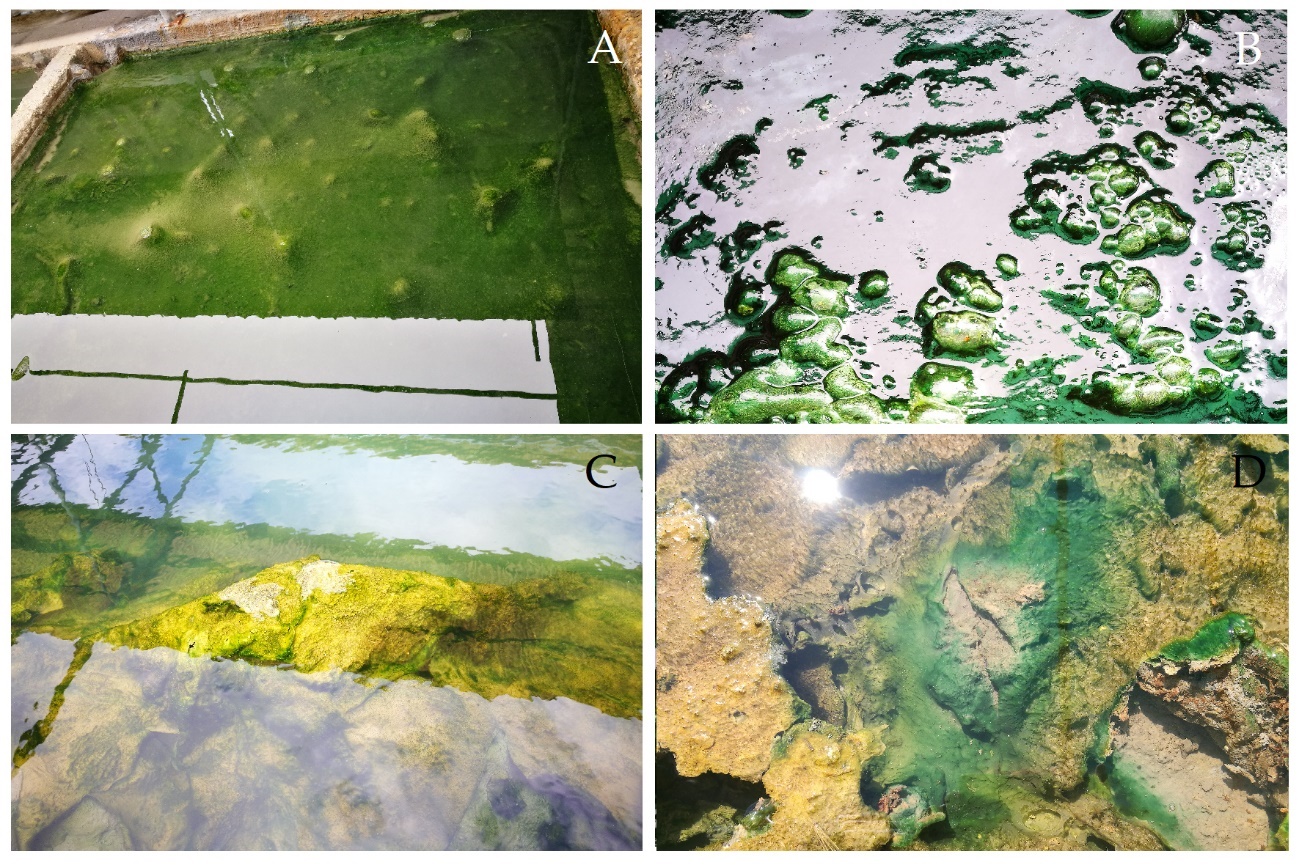


**Figure S7:** Green biofilms growing on the muds surface in maturation systems provided with protective roofs (A-B), and yellow biofilms from ponds or tanks exposed to solar radiation (C-D). In figure D a green face can be easily distinguished under the swallowed yellow biofilm.


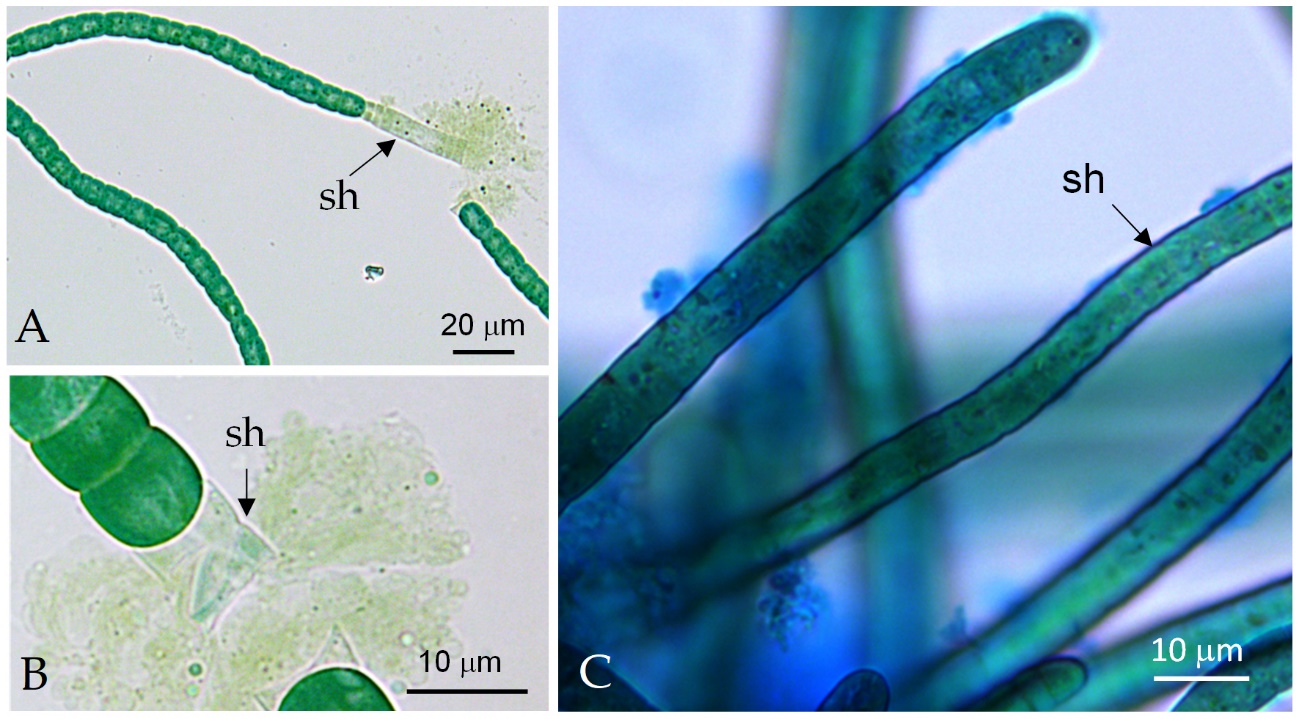


**Figure S8:** Micrographs of *Phormidium* sp. ETS-05 cultured at 40°C in BG11 medium. Light micrograph of the filaments (A-B) and filaments stained with Alcian blue (C) highlighting the sheath (sh).
